# Research Progress of Palladium-Plated Copper Bonding Wire in Microelectronic Packaging

**DOI:** 10.3390/mi14081538

**Published:** 2023-07-31

**Authors:** Yuemin Zhang, Haiyun Guo, Jun Cao, Xuefeng Wu, Hewei Jia, Andong Chang

**Affiliations:** School of Mechanical and Power Engineering, Henan Polytechnic University, Jiaozuo 454000, China; 212105010016@home.hpu.edu.cn (H.G.); cavan@hpu.edu.cn (J.C.); wuxuefeng@hpu.edu.cn (X.W.); jiahewei0305@163.com (H.J.); cadzmy@163.com (A.C.)

**Keywords:** microelectronic packaging, PdCu wires, distribution of Pd, intermetallic compound, reliability

## Abstract

Wire-bonding technology is the most commonly used chip interconnection technology in microelectronic packaging. Metal bonding wire is the key material for wire bonding and plays an important role in the reliability of electronic devices. In recent years, palladium-plated copper (PdCu) bonding wire has been widely used because of its low cost, good electrical and thermal conductivity, the fact that it is not easy to oxidize, and its high reliability. Therefore, it is necessary to review its research progress. In this paper, the preparation and application of palladium-plated copper bonding wire are reviewed. Firstly, the preparation methods of electroplating, electroless plating, and direct plating are introduced. Secondly, the factors affecting the distribution of Pd in free air balls and bonding interfaces, the effect of Pd on the formation and growth of intermetallic compounds in PdCu wire, stitch bond, and reliability of PdCu wire are summarized and analyzed in the application process. Finally, its development prospect is prospected. Hopefully, this review can help readers to have a comprehensive understanding of the preparation and application of palladium-plated copper bonding wires, and can accelerate the promotion of its application in more fields in the future.

## 1. Introduction

In microelectronic packaging, wire bonding is the most widely used and mature technology, which plays an important role in the performance of electronic devices, and metal-bonding wire is the key material of wire bonding. Gold-bonding wires have been dominant for a long time in the past, but in recent years, with the continuous rise of gold prices, it is urgent to find an alternative bonding wire with a lower cost. Copper (Cu) wire is becoming a standard wire-bonding material to replace gold wire in the semiconductor industry [1,2,3,4], especially in nanoelectronic packaging, because of its low price, excellent electrical and thermal conductivity, good strength and toughness, short heat-affected zone, and low necking failure rate [5,6,7,8,9].

However, the Cu wire has the following limitations in use: (1) oxidation of the wire surface; (2) the storage life before bonding; (3) stitch bondability; (4) the problem of increasing the cost of using mixed gas; (5) and long-term reliability. These problems limit the use of thin Cu wires in large-scale integration (LSI) packaging [10,11]. For example, using the Cu wire that has been oxidized on the surface for testing will cause the Cu ball to deviate from the ball in the burning ball, thereby reducing the bonding strength and affecting the reliability of the Cu wire.

Therefore, it is urgent to find a way to avoid Cu wire oxidation, prolong its storage life, and improve the reliability of Cu wire bonding. According to the literature, four kinds of metal thin coatings of Au, Ag, Pd, and Ni can prevent the oxidation of Cu wire [12,13,14]. The formation of spherical and high-quality free air ball (FAB) is the key requirement for the success of copper wire bonding. The melting point of Au and Ag is lower than that of Cu, and sharp balls will be formed during ball burning; Pd and Ni are precious metals, and their melting points are higher than that of Cu. However, it is found that the Ni-coated copper wire will form an eccentric ball during the burning of the ball. Only when Pd is plated can a good ball be formed [15,16]. Moreover, Pd has good ductility and plasticity, good adhesion to copper wire, a stable performance, and a high corrosion resistance in high-temperature and high-humidity environments [17]. These advantages of Pd promote the birth of PdCu wires. Pd coating not only solves the shortcomings of easy oxidation and high hardness of the Cu wire [18], but also prolongs its shelf life, broadens the bonding window of the second solder joint, and improves corrosion resistance and reliability [19,20,21,22,23,24]. Therefore, palladium-coated copper (PdCu) wire has been widely used in IC packaging, audio and video transmission lines, and other electronic devices.

The reliability of the PdCu wire is closely related to the quality of the Pd layer, the distribution position of Pd in FAB and the bonding interface, the growth of intermetallic compounds (IMCs), and the relationship between these three aspects is related to each other. The uniform and dense Pd layer on the surface of the Cu wire contributes to the relatively uniform distribution of Pd in FAB and the bonding interface. Pd slows down the growth of intermetallic compounds at the bonding interface, thereby improving the reliability of PdCu. How to obtain good Pd layer quality and then obtain PdCu wire with excellent performance, what are the factors affecting the distribution of Pd in FAB and the bonding interface, and what is the growth process and rate of Cu-Al intermetallic compounds in PdCu wire, these are the research hotspots in microelectronics packaging, and many scholars have conducted in-depth research on this. This paper analyzes and summarizes the above three aspects by referring to the literature.

## 2. Preparation of Palladium-Plated Copper (PdCu) Wire

At present, the preparation of PdCu wire in China mainly adopts electroplating, electroless plating, and direct plating technology. Electroplating and electroless plating technology is the earliest application technology. The preparation of palladium-plated copper (PdCu) wire by electroplating and electroless plating technology will produce harmful substances and pollute the environment [25]. Direct plating is a new Pd plating technology developed by researchers in recent years. It is simple to operate and does not produce harmful substances. It can be called “green Pd plating technology”.

### 2.1. Electroplating

**Figure 1:**
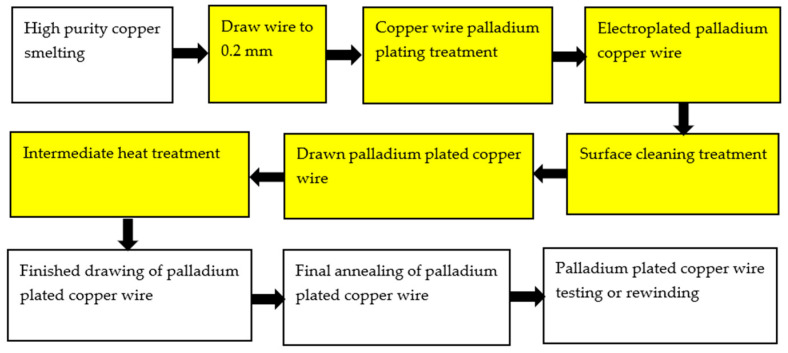
Palladium electroplating process for bonded copper wire [28]. Reproduced with permission from ref. [28]; published by Materials Science and Technology, 2015.

Electroplating is an important surface treatment technology. It uses electrochemical methods to process the surface of metal and non-metal products, so that various metal coatings can be obtained on the surface [26] to improve the corrosion resistance and oxidation resistance of materials. The plating solution is generally composed of metal salt, reducing agent, buffer and complexing agent, stabilizer, brightener, etc. The process parameters of electroplating mainly include pH value, temperature, time, and current density. At present, there are two kinds of palladium-plating solutions: ammonium salt type and phosphate type [27]. The preparation of PdCu wire by electroplating technology is to plate a thicker Pd layer on a thicker (Φ > 0.15 mm) Cu wire, and then to obtain the PdCu wire of the required specifications after drawing the PdCu wire, intermediate heat treatment, PdCu wire finished drawing, PdCu wire final annealing, PdCu wire test or rewinding, and other steps. However, due to the different plastic deformation properties of Cu and Pd during the drawing process, it is difficult to draw fine (Φ < 0.016 mm) PdCu wire [28]. Therefore, in some ultra-narrow spacing microelectronic packaging, it is difficult to prepare the required specifications of fine PdCu wire by electroplating technology, and the preparation process will produce harmful substances and pollute the environment. In the process of drawing, there may also be problems such as palladium layer shedding and uneven thickness of the palladium layer. Figure 1 shows the electroplating process [28].

### 2.2. Electroless Plating

Electroless plating is a plating method in which metal ions in the plating solution are reduced to metal and deposited on the surface of parts by means of a suitable reducing agent without impressed current. Electroless palladium plating can deposit palladium membrane with uniform thickness on the surface of the complex matrix. It is the most widely used method to prepare palladium membrane because of its low cost, simple equipment, easy operation, good uniformity, and compactness [29].

**Figure 2:**
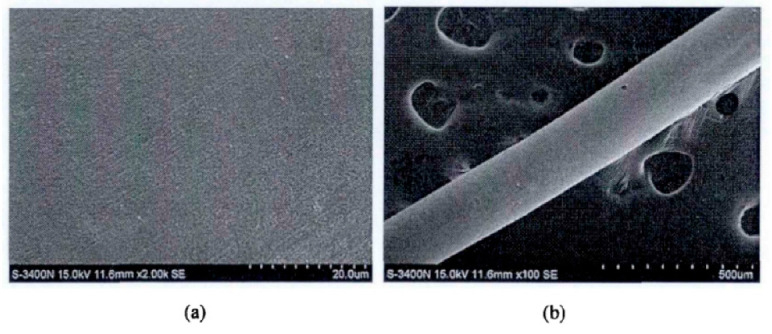
Surface morphology of palladium coating at different magnifications, (**a**): 2000 times and (**b**): 100 times [31]. Reproduced with permission of the master’s thesis advisor; Shanghai Institute of Technology, 2016.

Zhou et al. [30] developed a chemical method for the preparation of PdCu wire and applied for an invention patent. This method uses copper with a mass percentage greater than 99.95%, and then completes the preparation by melting and drawing, electroless palladium plating before annealing of the finished product, annealing, cleaning, rewinding and other steps. Compared with the traditional PdCu wire, the performance of the PdCu wire obtained by this preparation method has been improved, and the production process is safe, energy-saving, and simple. Zhang et al. [31] first studied electroless palladium plating on the surface of copper sheet, and then analyzed that due to the difference in the surface area of copper wire and copper sheet, the activation energy of palladium deposition on the surface of copper wire was small. By increasing the concentration of isopropanolamine to control the growth of the palladium particles, the formula composition and process suitable for electroless palladium plating on copper wire were obtained: PdCl_2_ 2 g/ L, NaH_2_PO_2_·H_2_O 12 g/L, 38% (mass fraction) HCl_4_ mL/L, 28% (mass fraction) NH_3_ H_2_O 160 mL/L, Bismuth nitrate 55 mg/L, auxiliary complexing agent isopropanolamine 35 mL/L pH 9.8, at a temperature of 52 °C and a duration of 35 min. The surface of the coating obtained by the formula composition and process is uniform and fine, and there is no delamination and crack, as shown in Figure 2.

PdCu wire is prepared by electroless plating. Although the production process has low cost, good uniformity, and compactness of the obtained palladium film, ultimately, the chemical solution is used, and the waste liquid will be produced in the preparation process, which will cause certain pollution to the environment and bring pressure to the ecological environment. The following focuses on a “green palladium plating process”.

### 2.3. Direct Plating

Direct palladium-plating technology, namely coating-palladium technology, is used to directly coat nano-palladium organic solution on the finished bonded copper wire, and deposit nano-palladium on the surface of copper wire by a heat-treatment process [28]. Therefore, the preparation of PdCu wire by direct-plating technology requires the preparation of PdCu wire blank line first, and then adjusting the coating process parameters, and finally obtaining the finished product.

**Figure 3:**
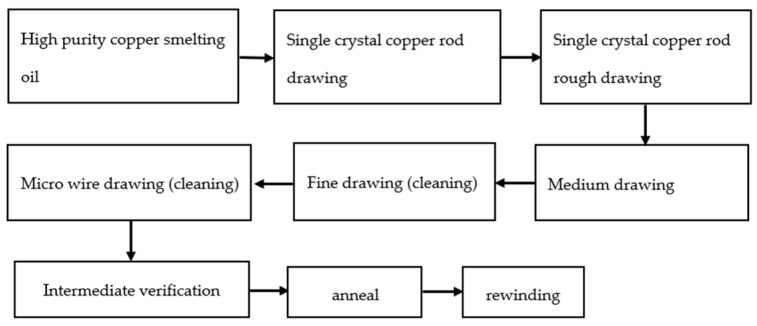
Bonded copper wire drawing process [33]. Reproduced with permission from ref. [33]; published by Foundry Technology, 2013.

Figure 3 shows the drawing process. The 6 N copper was drawn into 8 mm single-crystal copper rod in a single-crystal continuous casting furnace, and then the single-crystal copper rod was drawn into copper bonding wires of different specifications after rough drawing, medium drawing, fine drawing, and micro wire drawing [32].

**Figure 4:**
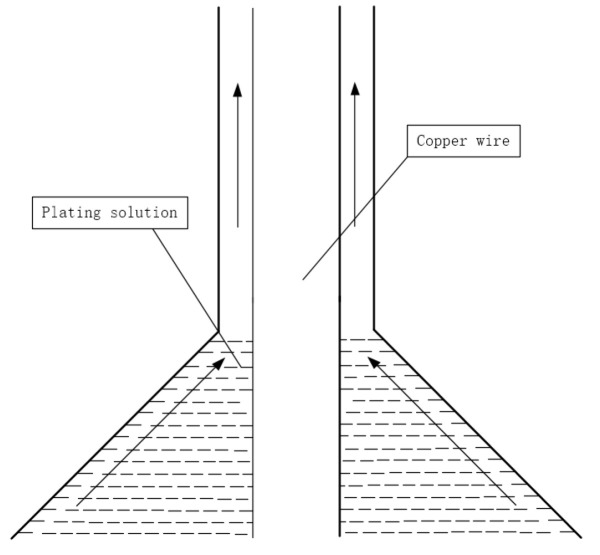
Coating schematic [33]. Reproduced with permission from ref. [33]; published by Foundry Technology, 2013.

Direct palladium-plating process [33]: (1) mold cleaning; (2) prepare the copper wire to be coated and wear the mold; (3) absorb the coating liquid with a medical needle tube without a rubber head; (4) according to the diameter of copper wire and the thickness of palladium layer, the speed of the bidirectional pump is set, and the bidirectional pump is opened; (5) when the speed of the pump reaches a predetermined value, start coating; (6) observe the coating color and adjust the temperature and speed appropriately. The coating schematic is shown in Figure 4.

**Figure 5:**
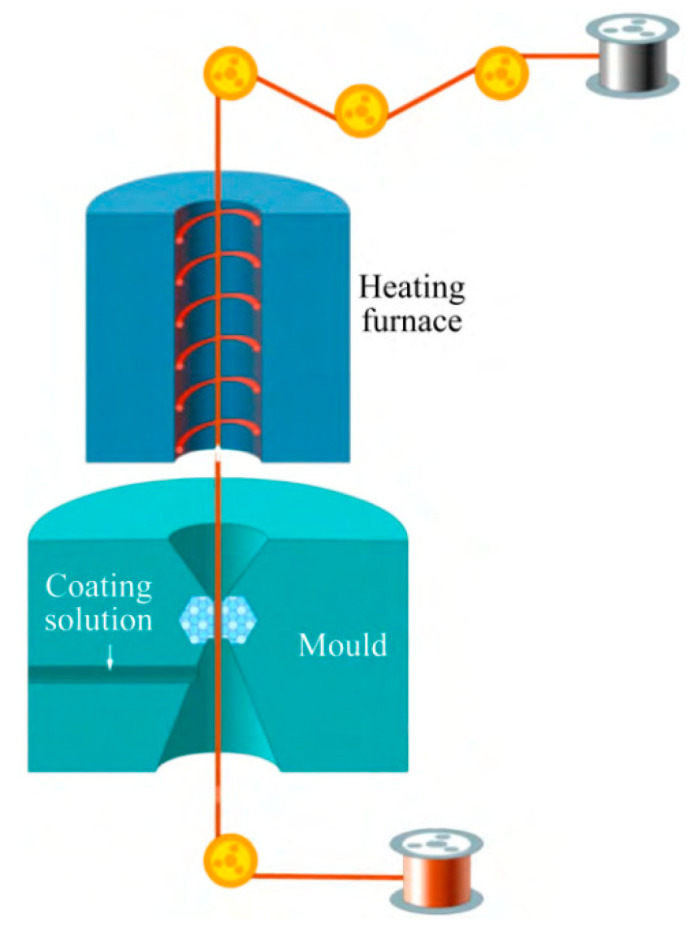
Principle of surface nano-coating process [34]. Reproduced with permission from ref. [34]; published by The Chinese Journal of Nonferrous Metals, 2020.

**Figure 6:**
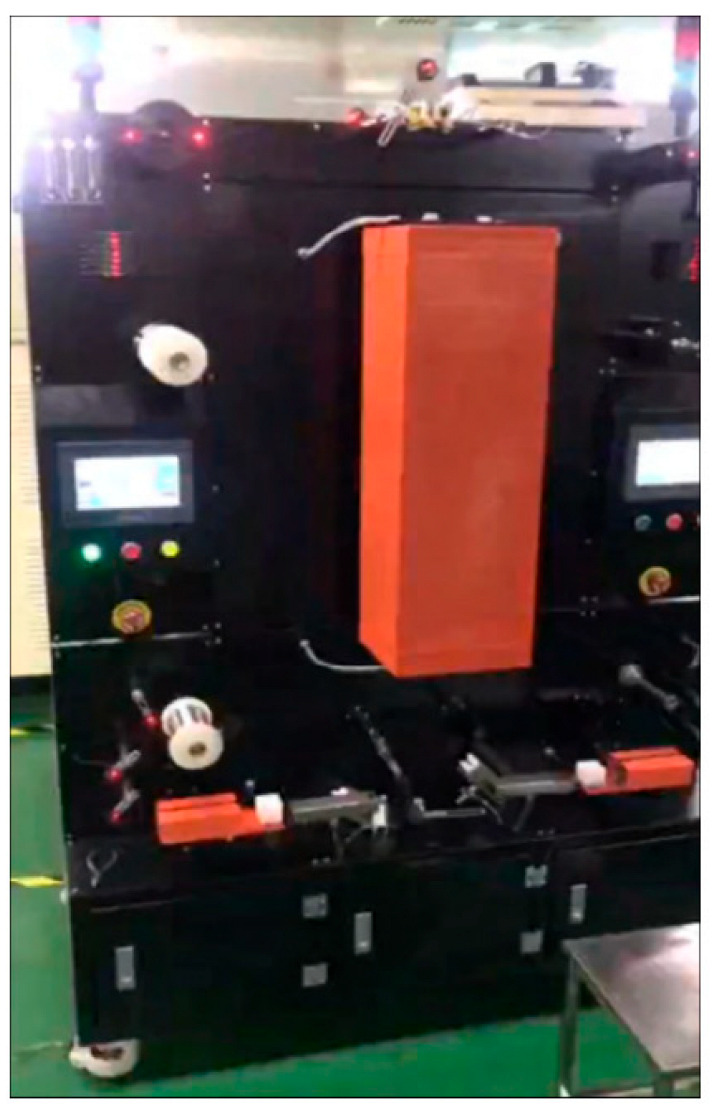
Nano-coating device for micro wire surface [34]. Reproduced with permission from ref. [34]; published by The Chinese Journal of Nonferrous Metals, 2020.

The principle of direct palladium-plating technology [34]: Firstly, by adding various chemical substances, the nano ultrafine powder is uniformly dispersed and suspended in the plating solution, and the plating solution has high wettability and stability. The surfactant in the plating solution is coated around the nanoparticles, and the dispersion between the nanoparticles is realized by the steric hindrance. Then, the drawn fine copper bonding wire is immersed in a specially prepared solution containing nano-metal powder. By drying the heating device and optimizing the heating deposition process parameters, a smooth and flat coating can be formed on the surface of the copper bonding wire. The effect of drying is to volatilize or decompose the solvent and other organic substances in the solution, leaving only the pure coating to adhere to the surface of the copper wire. Under the action of heat treatment, the coating completes the sintering process and is accompanied by grain growth and interface diffusion, thus forming a surface nano-coating with high interfacial bonding strength and brightness. The schematic diagram and device of surface nano-coating are shown in Figure 5 and Figure 6, respectively [34].

**Figure 7:**
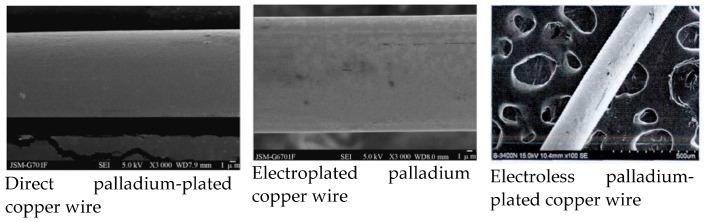
Comparison of surface morphology of PdCu wire [31,33]. Reproduced with permission of the master’s thesis advisor; Shanghai Institute of Technology, 2016; Reproduced with permission from ref. [33]; published by Foundry Technology, 2013.

The PdCu wire prepared by direct plating has good chemical stability, no cracking and peeling, excellent bonding performance, uniform coating, and a dense structure. Compared with electroplating and electroless plating, direct plating has the advantages of a simple process, low costs (approximately 11% of electroplating and electroless plating), environmental protection in the production process, no harmful substances, and a smooth and uniform coating surface [25,33]. From Figure 7, it can be seen that the surface quality of directly-plated palladium copper wire is better than that of electroplating and electroless plating. Therefore, in general, the performance (fracture force, elongation, etc.) of directly-plated palladium copper wire will be better than that of electroplating and electroless plating.

In summary, the fine copper bonding wire can be prepared by direct palladium-plating technology, and the prepared fine copper bonding wire has a good performance, stable chemical properties, a uniform palladium layer, and a good density. Direct palladium-plating technology is not only simple, but also produces no harmful substances in the preparation process, does not pollute the air and land, and is superior to electroplating and electroless plating.

## 3. The Distribution of Pd in FAB and Bonding Interface

In the application process of PdCu wire, due to the influence of test parameters and external environment, the distribution of Pd on FAB and bonding interface will be different, and the distribution of Pd has an important influence on the reliability of PdCu wire. Many scholars have studied the factors affecting the distribution of Pd and the specific distribution position of Pd.

**Figure 8:**
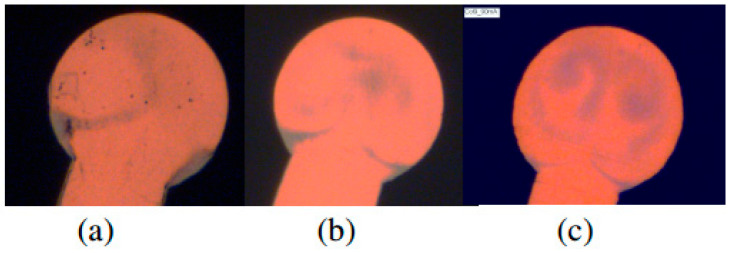
The optical images of FAB formed at EFO currents (**a**) 30 mA, (**b**) 60 mA, and (**c**) 90 mA showing different distributions of Pd [35]. Copyright, 2010, IEEE.

It is found that there are many factors affecting the distribution of Pd, such as Electronic Flame-Off (EFO) discharge current, EFO discharge time, EFO discharge electrode position, Pd layer thickness, environmental factors of aging test, PdCu wire type, and so on. After consulting the literature, it is found that the distribution position of Pd on the FAB has a great relationship with the EFO discharge current, and scholars have conducted in-depth research on it. Yauw et al. [35] used 0.8 mil PdCu wire and set three EFO discharge currents (30, 60, and 90 mA) to study the distribution of Pd on FAB. The results showed that under a low EFO discharge current, there was a thick Pd layer on the surface of FAB, while a small amount of Pd was distributed at the core of FAB (Figure 8a). At a higher EFO discharge current, Cu melts rapidly resulting in turbulent effects, and more Pd is mixed into the core of the FAB (Figure 8b,c).

**Figure 9:**
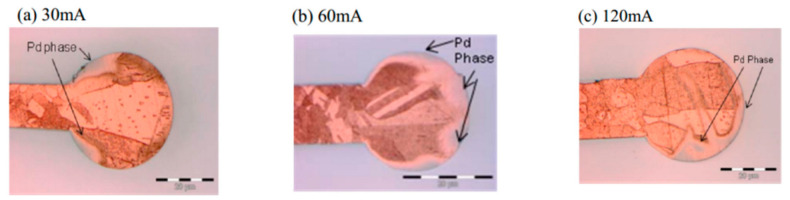
Pd distribution on FAB at 30 (**a**), 60 (**b**) and 120 (**c**) mA EFO current [37]. Copyright, 2010, IEEE.

Tang et al. [36] used two types of PdCu wires with a diameter of 0.8 mil, and also set three EFO discharge currents (30, 60, and 90 mA) to test. FAB was corroded with ferric chloride (FeCl_3_) solution. It was found that increasing the EFO discharge current can make Pd symmetrically distributed on the surface of FAB; in this experiment, a Pd layer with a thickness of 14 nm can be obtained on a FAB with a diameter of 34 μm by using a 90 mA EFO discharge current. Clauberg et al. [37] studied the distribution of Pd on FAB by setting three EFO discharge currents (30, 60, and 120 mA) with 20 μm PdCu wire. The results show that at 30 mA current, the heating rate of the tip of PdCu wire is slow, and most of Pd stays on the surface of the wire. Cu flows out from the Pd shell and forms FAB due to the surface tension. Therefore, Pd is mainly distributed in the neck of FAB (Figure 9a). At a higher EFO discharge current, the tip of the PdCu wire is heated very fast, causing a turbulence effect, so Pd randomly exists in a general eddy current mode in the FAB (Figure 9b,c). The dark gray area in Figure 9 is the Pd coverage area.

**Figure 10:**
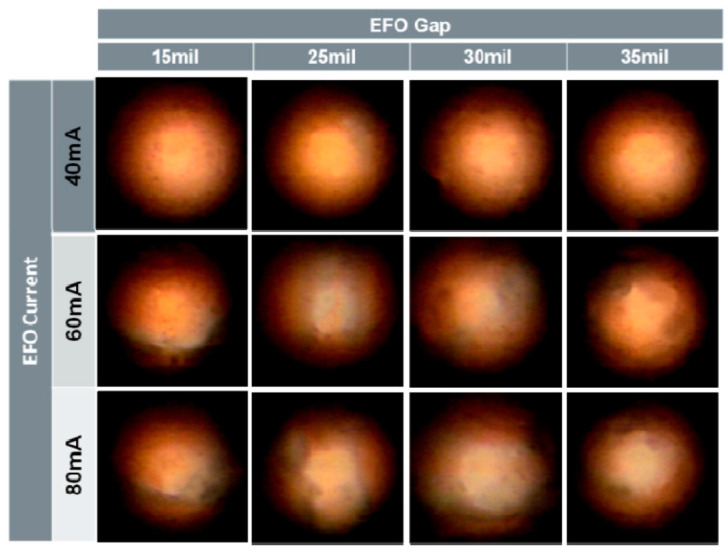
Optical Image of FAB Surface as a Function of EFO Gap and EFO Current [20]. Copyright, 2011, IEEE.

Cheng et al. [38] studied the effect of different EFO discharge currents (50, 70, and 90 mA) on the distribution of Pd on FAB using 18 μm PdCu wire (99.99 wt. % purity of copper, 0.1 μm thickness of Pd layer). The study found that different EFO discharge current settings lead to full or partial coverage of Pd on FAB and will also directly affect the distribution of Pd at the bonding interface. Lim et al. [39,40] used 0.6 mil PdCu wires to conduct experiments under low, medium, and high EFO discharge current settings. Then, a FEI dual beam focused ion beam (FIB) system was used to analyze the cross-section of FAB samples. The study found that under low EFO discharge current settings, Pd remained on the surface of FAB; under the discharge current setting of medium EFO, the rich Pd phase is mainly concentrated in the neck region of FAB; under the high EFO discharge current setting, some Pd will be unevenly injected into the FAB due to the turbulence of Cu, resulting in the formation of Pd-rich trajectories within the FAB. Stephan et al. [20] used a 15 μm PdCu wire to test under different EFO discharge currents (40, 60, and 80 mA), and then studied the distribution of Pd on the FAB surface by optical imaging of the FAB surface through the bright field imaging technique. From Figure 10, it can be seen that as the EFO discharge current increases, the Pd coverage on the FAB projection surface also increases, and the high EFO current can move the Pd enrichment phase to the top of the FAB. When bonding with the Al pad, a Pd shell is formed.

The discharge time of EFO has an effect on the distribution of Pd on FAB. Du et al. [41] analyzed that the density of Pd was greater than that of Cu, so Pd would move downward and solidify with the increase of time during the solidification process of FAB. Therefore, a longer EFO discharge time will lead to a larger Pd coverage on the FAB surface.

**Figure 11:**
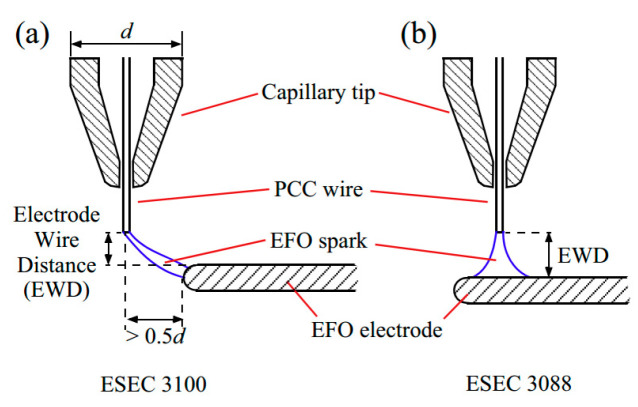
(**a**) fixed electrode; (**b**) moving electrode [42]. Copyright, 2015, Elsevier.

**Figure 12:**
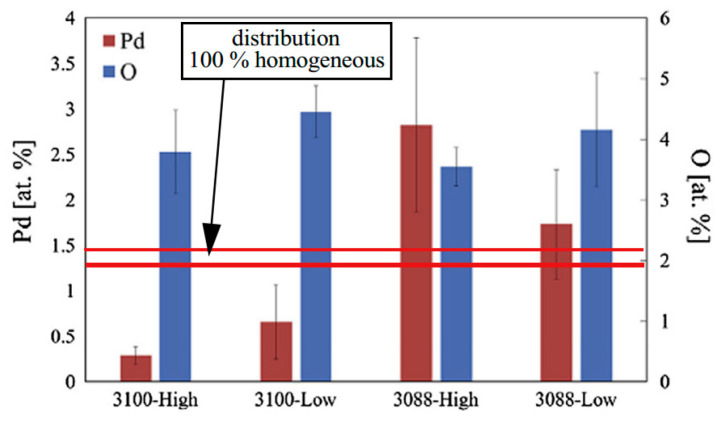
Pd and O content (at. %) on FAB surface at 3100 (fixed electrode) and 3088 (removable electrode) [42]. Copyright, 2015, Elsevier.

**Figure 13:**
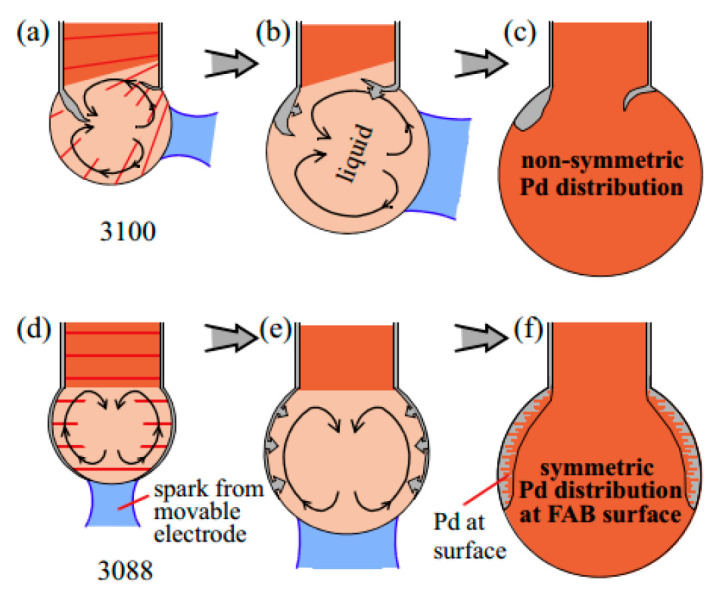
Convection patterns and resulting Pd distributions at fixed (**a**–**c**) and movable (**d**–**f**) electrodes in the early (**a**,**d**), middle (**b**,**e**), and final (**c**,**f**) stages of FAB formation [42]. Copyright, 2015, Elsevier.

After analysis, some scholars believe that the distribution of Pd is related to the position of EFO discharge electrode, and the energy generated by different positions of EFO discharge electrode is different in the distribution of FAB, which leads to the difference in Pd distribution. Ly et al. [42] used PdCu wire with a purity of 4 N (99.99 wt. %) and a diameter of 20 μm (the thickness of Pd layer is approximately 70–90 nm) to study the distribution of Pd by fixed electrode and movable electrode (Figure 11) and set high and low EFO discharge current. From the Energy Dispersive X-Ray Spectroscopy (EDX) elemental analysis near the equator of FAB (Figure 12), it can be seen that the Pd content on the surface of the fixed electrode FAB is significantly less than that on the surface of the moving electrode FAB. They also analyzed a possible reason for the difference in Pd distribution between the fixed electrode and the movable electrode: the different positions of the EFO electrode lead to different directions of the spark plasma approaching the wire end. For the fixed electrode, the plasma heats the end of the wire from the side, as shown in Figure 13a,b, which results in a lower surface temperature on the opposite side, resulting in less melting and diffusion of the Pd layer on that side; for the movable electrode, its spark produces a more axisymmetric temperature distribution (Figure 13d), so the Pd concentration on the FAB surface is relatively high.

The distribution of Pd on FAB is related to the solid solution (Cu, Pd). Pd and Cu have the same crystal structure (Face Center Cubic, FCC) and similar crystal constants [43]. According to the Cu–Pd phase diagram [44], FCC continuous solid solution (Cu, Pd) exists above 598 °C. Because the melting point of Cu is 1083 °C, when the ball is burned, the copper bonding wire melts, and Pd is likely to dissolve in the Cu matrix to form a solid solution. Lim et al. [40] found that the heat energy generated by EFO sparks is mostly used to provide the melting of Cu wire, Pd will not completely melt, Pd and Cu will form a solid solution, but will not form a uniform solid solution. Yeung et al. [45] found that in the actual melting process of PdCu wire, the heating duration is too short, and Pd cannot be completely dissolved into the Cu matrix before FAB cooling. According to the solid solubility of the two metals, Pd and Cu can form a solid solution, but will not form a total solid solution.

**Figure 14:**
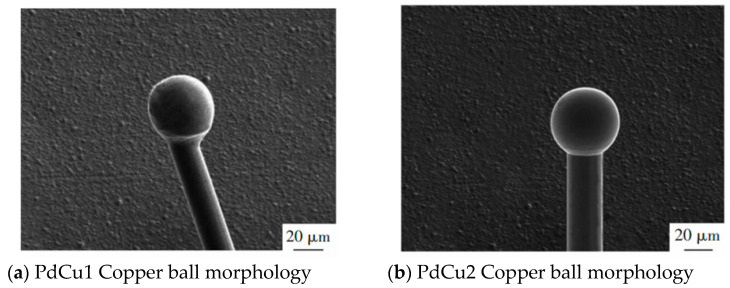
Morphology of PdCu1 wire and PdCu2 wire copper balls [46].

**Figure 15:**
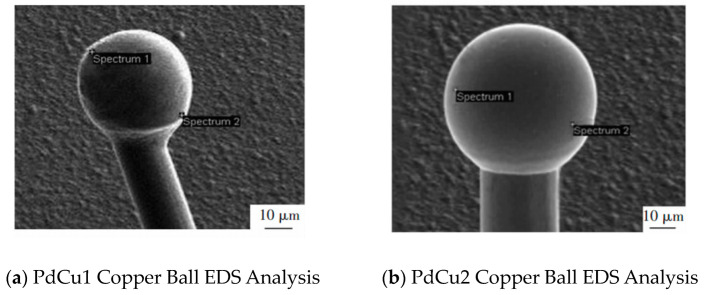
EDS analysis of PdCu1 wire and PdCu2 wire copper balls [46].

The distribution of Pd on FAB is related to the thickness of Pd layer. Cao et al. [46] used PdCu wires with a diameter of 20 μm and different Pd layer thicknesses (PdCu1: Pd layer thickness 35–50 nm and PdCu2: Pd layer thickness 85–100 nm) to conduct ball burning experiments to observe the morphology of FAB, and analyzed the Pd distribution on FAB using EDS. The results are shown in Figure 14 and Figure 15: PdCu wires with a Pd layer thickness that is too small can cause FAB spheroidization and uneven Pd distribution during EFO discharge; it is also concluded that for a 20 μm PdCu wire, the thickness of the Pd layer should not be less than 95 nm, which means that the thickness of the Pd layer should not be less than 0.475% of the wire diameter.

**Figure 16:**
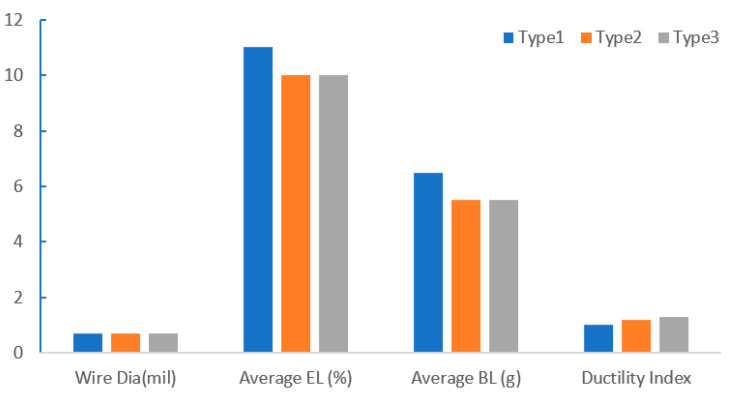
Properties of different types of PdCu [47].

Under the same EFO discharge current, the distribution of Pd on FAB is related to the type of PdCu wire. Tang et al. [47] studied the distribution of Pd on three different types of PdCu wires (Figure 16) under the conditions of EFO discharge current of 60 mA and EFO discharge time of 150 us. The results show that the distribution of Pd on different types of PdCu wires is different. Compared with type 1 PdCu wire, Pd is more completely distributed on the FAB of type 2 and type 3 PdCu wires. For these three PdCu wires, the Pd content in the neck was the highest after the formation of FAB, and then gradually decreased until the maximum diameter of FAB.

**Figure 17:**
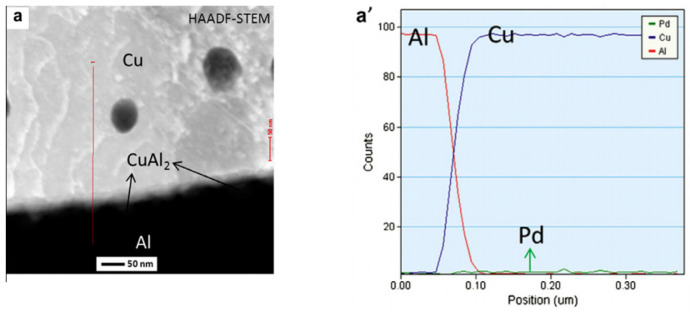

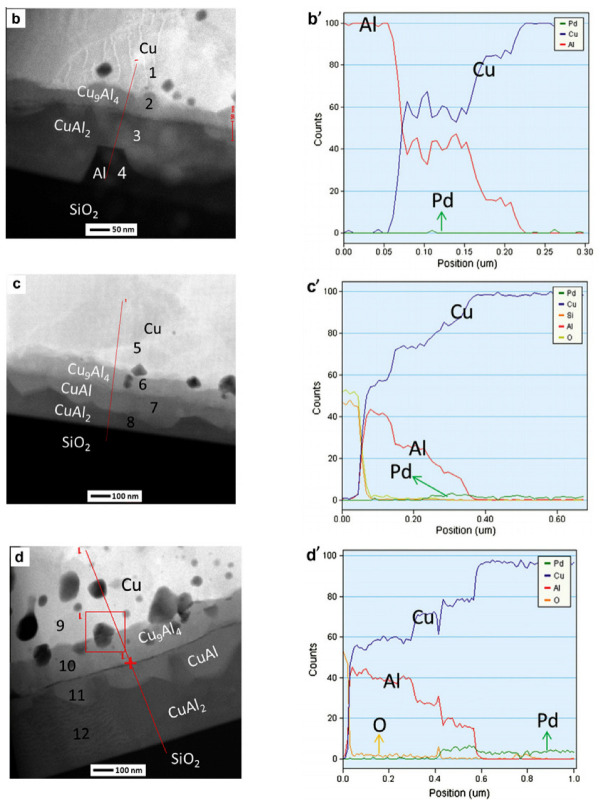
Evolution of Pd at the central interface of PdCu wire bonds during aging at 175 °C: (**a**,**a**’) as-bonded; (**b**,**b**’) 24 h; (**c**,**c**’) 48 h; (**d**,**d**’) 168 h. EDX line scans from Al pad to Cu ball [19]. Copyright, 2013, Elsevier.

**Figure 18:**
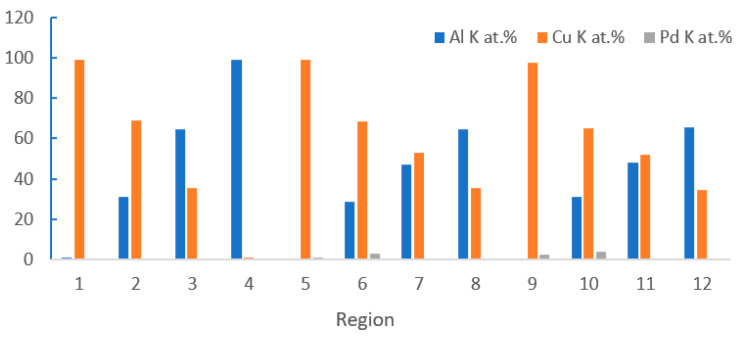
STEM-EDX results for regions 1–4 in Figure 17b, regions 5–8 in Figure 17c, and regions 9–12 in Figure 17d [19].

**Figure 19:**
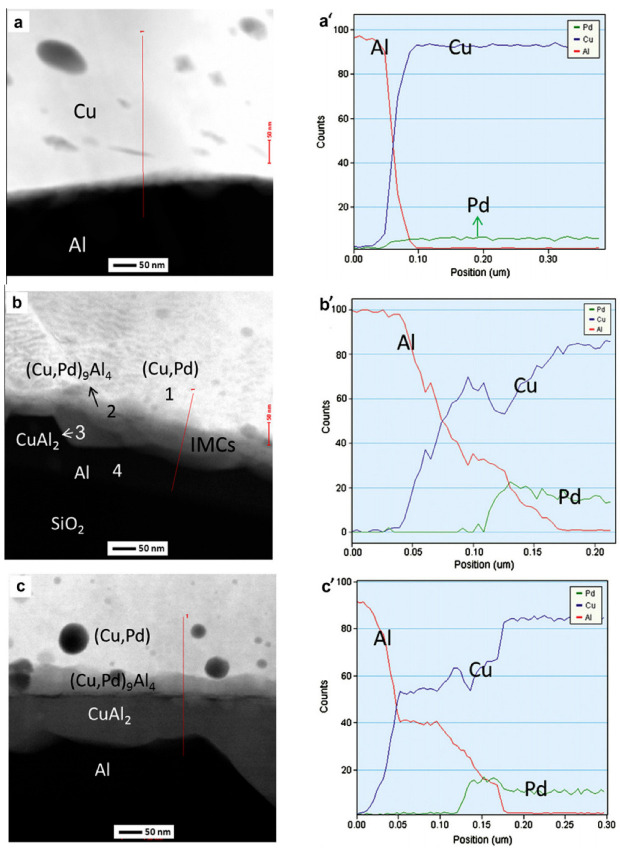

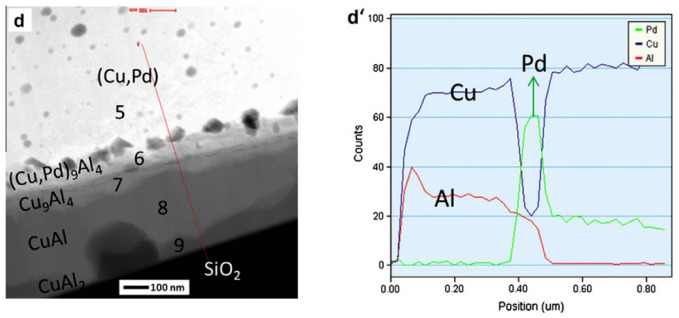
Evolution of Pd at the peripheral interface of PdCu wire bonds during aging at 175 °C: (**a**,**a’**) as-bonded; (**b**,**b’**) 24 h; (**c**,**c’**) 48 h; (**d**,**d’**) 168 h. EDX line scans from Al pad to Cu ball [19]. Copyright, 2013, Elsevier.

**Figure 20:**
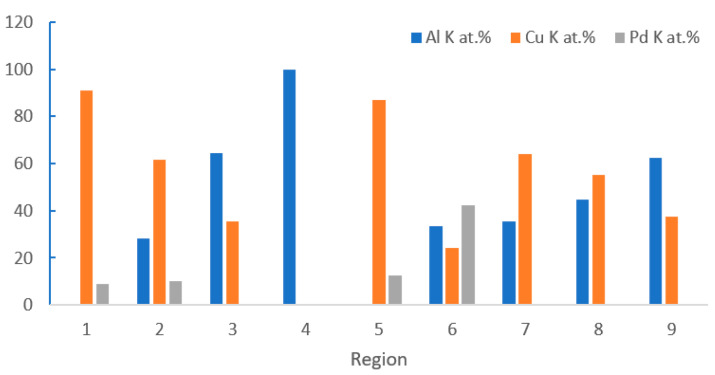
STEM-EDX results for regions 1–4 in Figure 19b, and regions 5–9 in Figure 19d [19].

The distribution of Pd on FAB largely determines the position of Pd at the bonding interface. With the vertex of FAB as the center, approximately 44.5% of the spherical surface area around it will participate in the formation of the bonding interface [48], so Pd in this area will exist at the bonding interface. However, the position of Pd at the bonding interface is not immutable. In the aging test, with the increase of aging time, Pd will re-aggregate and re-locate. Xu et al. [19] used a 0.7 mil PdCu wire to test. The selected samples were aged in air at 175 °C for 168 h. The distribution of Pd in the central and peripheral regions of the bonding interface was studied by transmission electron microscopy (TEM) combined with energy dispersive X-ray spectroscopy (EDX). The distribution of Pd in the central region: In the bonding state, EDX line scanning from the Al pad to the ball shows that there is no Pd concentration at the central interface (Figure 17a’) and the Cu-Al diffusion zone is less than 50 nm. The EDX line scan of the center interface after aging at 175 °C for 24 h shows that there are two layers of IMCs (Cu9Al4 is adjacent to Cu and CuAl2 is adjacent to Al) and no Pd (Figure 17b,b’ and Figure 18); after 48 h of aging, the Al layer is completely consumed, and three layers of IMCs are formed (Figure 17c). EDX results show that the Pd content in the central interface region is less than 3 at. % (Figure 17c’). After 168 h of aging, EDX results showed that a limited amount of Pd (less than 5 at. %) was detected in the Cu_9_Al_4_ layer and the Cu ball, but not in the CuAl or CuAl_2_ layers (Figure 17d,d’ and Figure 18). The distribution of Pd in the peripheral region: A small amount of Pd (2 at. %) can be identified at the peripheral interface of the bonding state (Figure 19a,a’); after 24 h of aging, the amount of Pd increased to 10 at. % and was located in the Cu_9_Al_4_ layer and Cu ball, but not in the CuAl_2_ layer (Figure 19b,b’ and Figure 20). The Pd atom in Cu_9_Al_4_ partially replaced the Cu atom, so it can be rewritten as (Cu, Pd)_9_Al_4_; after 48 h of aging, the EDX line scan showed that the Pd concentration in the (Cu, Pd)_9_Al_4_ layer was the highest, followed by the (Cu, Pd) ball, but the CuAl_2_ layer did not contain any Pd (Figure 19c,c’). After 168h of aging, a Pd-rich (Cu, Pd)_9_Al_4_ layer (42 at. % Pd) with a thickness of less than 100nm, close to that of Cu, appeared (Figure 19d,d’). With aging, the Pd concentration in (Cu, Pd)_9_Al_4_ increases, the Cu concentration decreases, and the sum of Pd and Cu concentrations is almost constant. It is worth noting that Pd does not appear in all IMCs layers. It can only replace Cu in Cu_9_Al_4_, but not Cu in CuAl or CuAl_2_, so it can be concluded that Pd accumulates and relocates during aging, especially in the peripheral region of the bonding interface. Qin et al. [43] also used a 0.7 mil PdCu wire to test. The selected sample was aged at 175 °C for 168 h, and then the high-resolution transmission electron microscope (HRTEM) with Energy Dispersive X-Ray Spectroscopy (EDX) was used to observe the behavior of Pd during the aging process. It was also found that the behavior of Pd at the central interface and the peripheral interface was different. After aging at 175 °C for 168 h, a small amount of Pd (2.5–3.0 at. %) was detected in the central region; however, a Pd-rich layer with a thickness of less than 100 nm is formed in the peripheral region.

In summary, there are many factors that affect the distribution of Pd on the FAB, the most significant of which is the EFO discharge current. The distribution of Pd on FAB has a great influence on the distribution of Pd at the bonding interface, and Pd will re-aggregate and locate at the bonding interface with the increase of aging time. According to the literature, Pd acts as a barrier layer at the bonding interface, blocking the diffusion of Cu and Al atoms, slowing down the growth of Cu-Al IMC, and thus improving the reliability of PdCu wire.

## 4. The Effect of Pd on Intermetallic Compounds (IMCs) and Reliability Analysis

An appropriate amount of interfacial IMCs formation in ultrasonic or thermal ultrasonic wire bonding of dissimilar metals will increase the bonding strength, but too much or too little IMCs formation may lead to a decrease in bonding performance [49,50].

The reliability in electronic packaging refers to the ability of the product to complete the specified functions under the specified conditions and within the specified time. The reliability test refers to the acceleration of the product by simulating the temperature change, humidity change, and force change in the real environment. The service status in the actual use environment verifies and obtains the quality status of the product [48].

The mechanical reliability of wire bonding in microelectronic packaging depends largely on the formation and growth of intermetallic compounds (IMCs) between the bonding ball and the pad, so intermetallic compounds are necessary for successful bonding [51,52].

**Figure 21:**
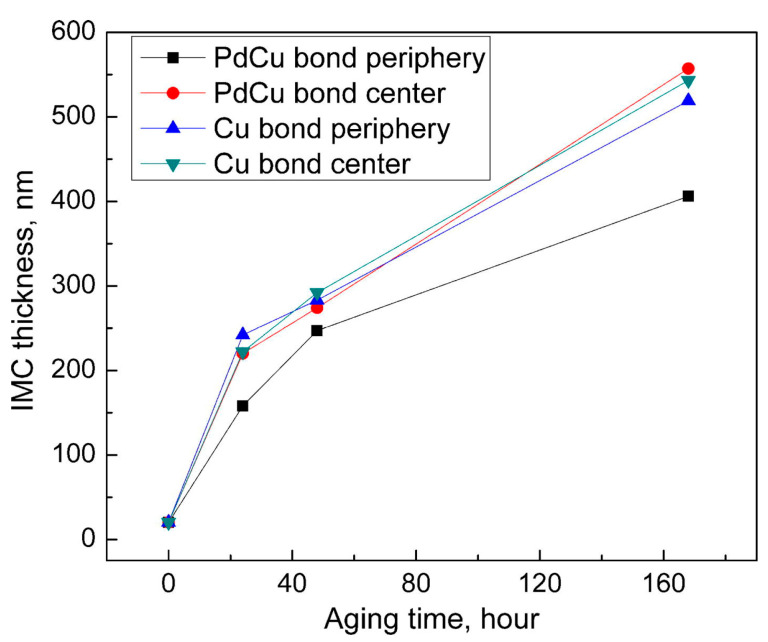
Comparison of IMC thickness between PdCu wire and bare Cu wire after aging at 175 °C [19]. Copyright, 2013, Elsevier.

Scholars have conducted in-depth research on the growth rate of Cu-Al IMCs and the effect of Pd on its growth rate. Through the analysis of atomic properties, it is found that compared with Au the atomic radius difference between Cu and Al is larger, and the electronegativity of Cu is smaller. Therefore, the formation rate of Cu-Al IMCs is much slower than that of Au-Al, approximately 1/10 of Au-Al, and the thickness is very thin [53,54,55]. Early studies have shown that the Cu-Al IMCs of bare Cu wire is hard and brittle [56,57,58], but in the Cu-Al IMCs of PdCu wire, because the electronegativity difference between Pd and Cu is very small (Pd is 2.2, Cu is 1.9) [59,60], it is easy to form a solid solution with a face-centered cubic structure, so the Cu-Al IMCs of PdCu wire has better ductility and a slower growth rate [61]. It has been reported that Cu-Al IMCs in PdCu wire bonding is thinner than that in bare Cu wire bonding [62], because Pd can slow down the growth rate of IMCs in PdCu wire bonding [43,63,64,65,66]. Xu et al. [19] compared the IMCs thickness between the bonding of bare Cu wire and PdCu wire at the aging temperature of 175 °C. The results are shown in Figure 21. Because during the aging process, Pd will re-diffuse and gather in the peripheral region of the bonding interface, the Pd in the central region is very limited. Therefore, as the aging time increases, the IMC’s thickness in the peripheral region of PdCu wire becomes thinner, and the IMC’s thickness in the central region is similar to that of bare Cu wire. Some scholars have found that the reason why Pd can slow down the growth rate of Cu-Al IMCs is that the Pd-rich layer at the bonding interface acts as a diffusion barrier between Cu atoms and Al atoms [64,67,68,69]. In addition, studies have shown that Pd at the bonding interface helps to maintain bonding strength after aging tests [64,70].

The formation of stable IMC is one of the main factors indicating reliability [54]. In the process of metal interdiffusion, the growth rate of the intermediate phase follows the parabolic rate law, which can be described by the empirical equation [55,71,72,73,74]: δ = (Kt)^(1⁄2), where δ is the thickness of the IMC’s layer, K is the reaction rate of IMC formation, t is the aging time, and Lim et al. [64] also confirmed the accuracy of this equation through experiments. Hang et al. [49] used a Cu wire with a diameter of 50.4 μm and a purity of 99.99 wt. % to study the growth of Cu-Al IMCs in a high-temperature aging test at 250 °C for 196 h. The formation rate of IMCs is approximately 6.2 ± 1.7 × 10^−14^ cm^2^/s.

**Figure 22:**
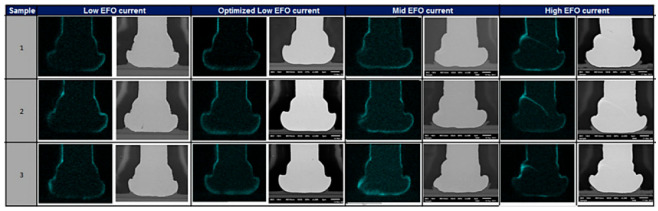
EDX elemental diagram shows the Pd distribution of the bonding ball under various EFO current settings [40]. Copyright, 2014, Elsevier.

**Figure 23:**
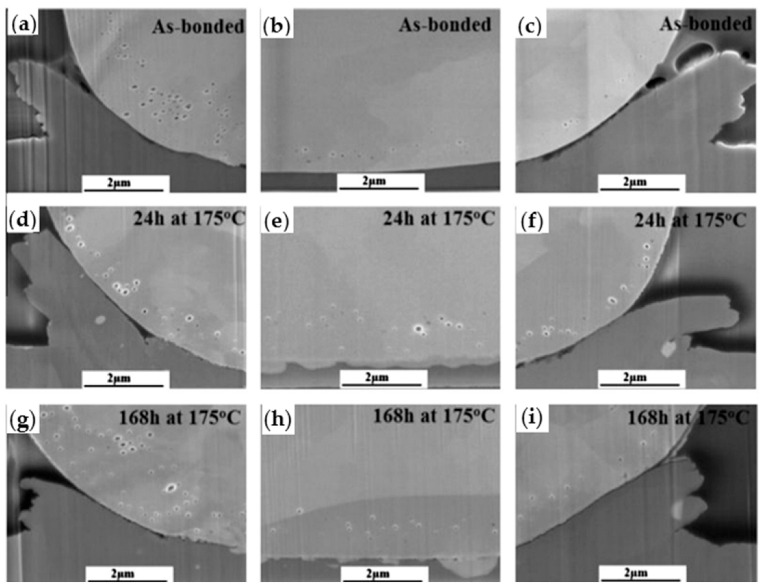
Kirkendall voids at the bonding interface at different annealing times (**a**,**d**,**g**) left peripheral region, (**b**,**e**,**h**) central region, (**c**,**f**,**i**) right peripheral region [40]. Copyright, 2014, Elsevier.

**Figure 24:**
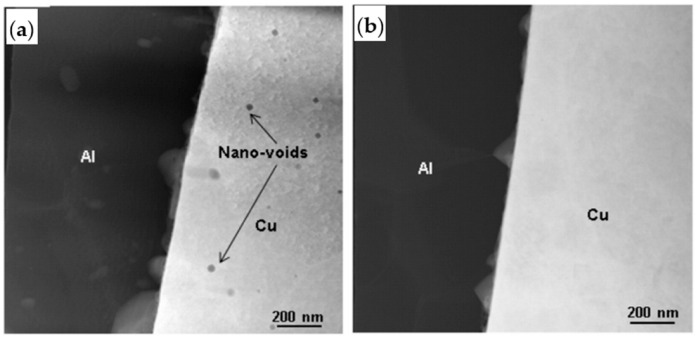
HAADF STEM images of (**a**) the Pd-Cu bond containing Pd showing nano-voids in the Cu (dark spots) and (**b**) the Pd-Cu bond with no Pd [64]. Copyright, 2016, Elsevier.

During the growth of IMC, Kirkendall voids are undesirable because it is generally believed that Kirkendall voids weaken the strength of wire bonding [51,53,75] and eventually lead to bonding failure. The occurrence of Kirkendall voids is closely related to Pd [76]. Lim et al. [64] found Kirkendall voids where there was Pd at the bonding interface of the PdCu wire, and no Kirkendall voids were found where there was no Pd. Lim et al. [40] studied the relationship between the formation of Kirkendall voids and the presence of Pd by setting low, medium, and high EFO discharge currents for bonding experiments, and then storing them in air at 175 °C for 168 h. As shown in Figure 22, for low EFO discharge current, no visible Pd-rich layer is found at the bonding interface, and a visible Pd-rich layer is found at the bonding interface with optimized low EFO discharge current; for the medium EFO discharge current, the presence of Pd was found at the bonding interface; for high EFO discharge current, some Pd injection was observed in the bonding ball. The high-temperature storage bonding interface of the test sample under the optimized low EFO discharge current setting is shown in Figure 23. Kirkendall voids were found in the central and peripheral areas of the bonding interface, so the formation of Kirkendall voids was closely related to the presence of Pd, and they believed that Kirkendall voids were formed by the volume shrinkage of FAB during the curing process due to the different cooling rates of Cu and Pd. Lim et al. [64] used 0.6 mil Cu wire and PdCu wire for comparative study. The bonding interface image is shown in Figure 24. The study found that there are no Kirkendall voids at the bonding interface of the Cu wire. There are many Kirkendall voids at the bonding interface of PdCu wire, and EDX shows that Pd exists in these areas. Therefore, Kirkendall voids are closely related to the presence of Pd.

Scholars have found through research that the formation of Kirkendall voids is also related to the growth of IMCs, as the volume of IMCs decreases due to phase transition, resulting in the formation of Kirkendall voids [77,78]. Lee et al. [66] stored the bonded samples of PdCu wires with a diameter of 20 μm at high temperature for 14,098 h and found that cracks appeared at the periphery of the bonding interface when the Cu Al IMCs phase transformed from Cu rich to Al rich. They believed that the cracks were caused by volume changes during the IMCs phase transition.

**Figure 25:**
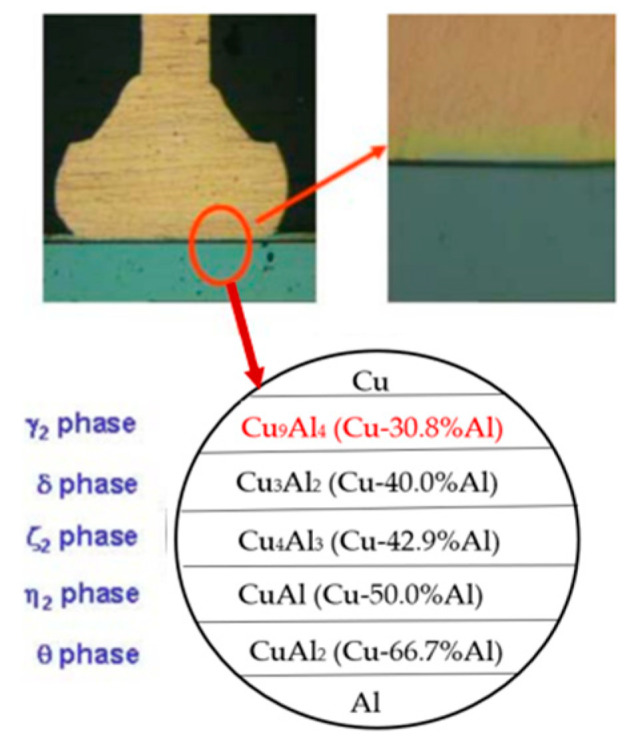
IMCs type of Cu-Al bonding interface [54]. Copyright, 2010, IEEE.

**Figure 26:**
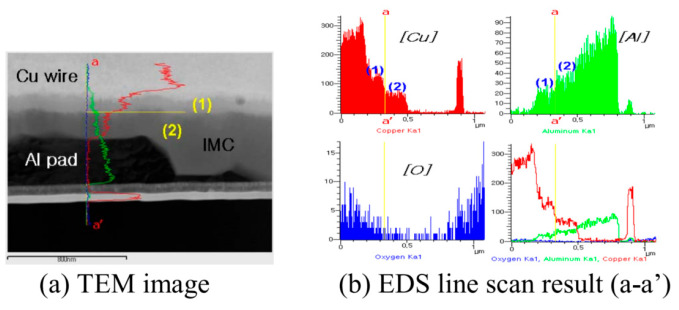
Formation of IMC at Cu-Al bonding interface [54]. Copyright, 2010, IEEE.

According to the binary phase diagram of the Cu-Al system, it can be observed that five types of IMCs can be formed at 150–300 °C, namely Cu_9_Al_4_, Cu_3_Al_2_, Cu_4_Al_3_, CuAl, and CuAl_2_ [79,80,81], among which Cu_9_Al_4_, CuAl, and CuAl_2_ compounds are relatively stable [82]. Stephan et al. [20] found that in the Cu-Al system, the formation of IMCs starts from the most Al-rich IMCs (CuAl_2_), followed by CuAl, and finally forms Cu-rich IMCs (Cu_9_Al_4_). Several scholars have also found that in bare Cu wires, the Cu-Al IMC phases are usually Cu_9_Al_4_ and CuAl_2_, with a relatively small amount of CuAl present [49,83,84,85,86]. It has been found that discontinuous island-like CuAl_2_ IMC particles have been formed in the Cu-Al bonding state [87,88,89,90]. After 24 h and 168 h of annealing time, two layers of IMCs are formed, one near the Cu bond (Cu_9_Al_4_) and the other adjacent to the Al pad (CuAl_2_) [91]. Kim et al. [54] analyzed the IMCs’ composition of the Cu wire and Al pad bonding interface. The IMCs’ composition can be divided into five types (Figure 25), but only Cu_9_Al_4_ and CuAl_2_ are usually observed. Some scholars have confirmed that Cu_9_Al_4_ and CuAl_2_ are indeed the main IMC products through experiments [49,92]. The main distribution positions of Cu_9_Al_4_ and CuAl_2_ in the bonding interface are shown in Figure 26. In Figure 26a, the IMCs are formed between the Cu wire and the Al pad [54], and region (1) is considered to be a Cu_9_Al_4_ layer with higher Cu and lower Al IMC; region (2) is considered to be a CuAl_2_ layer with lower Cu and higher Al IMC. Figure 26b is the EDS scan results of regions (1) and (2).

Early studies have found that in the Cu-Al IMCs system, the phase most prone to failure is the Cu-rich phase (mainly Cu_9_Al_4_) near the bottom of the Cu bonding ball [93,94,95,96]. Kim et al. [55] used a bare Cu wire with a diameter of 2.0 mil to test, and then performed microscopic XRD analysis on the bonding interface. It was found that the main IMC phase on the broken bond was Cu_9_Al_4_. In aging tests, in general, the formation of IMCs depends not only on time and temperature, but also on the size of the available material volume at the bonding interface. In the case of Cu wire bonding, the IMC’s volume is provided by the Cu bonding ball and Al pad metallization. Therefore, it is very important to determine the initial thickness of the Al pad and the residual thickness of the Al pad after bonding. This amount will determine the formation of Cu-Al IMCs during the aging process. The more Al is available, the later the Al is completely consumed, and the more Cu-Al IMC is formed [20]. Chen et al. [77] conducted high-temperature storage (HTS) on samples with good bare Cu wire bonding and found that CuAl_2_ and Cu_9_Al_4_ were present in the good samples, while CuAl_2_ and CuAl were present in the damaged samples. The Al layer was depleted in the damaged samples, and the layer with Cu_9_Al_4_ on the Cu side passed through HTST without consuming all of the Al. Therefore, they believe that the thickness of the Al was too small, resulting in failure to form a large amount of IMCs when Al was completely consumed, resulting in sample damage.

**Figure 27:**
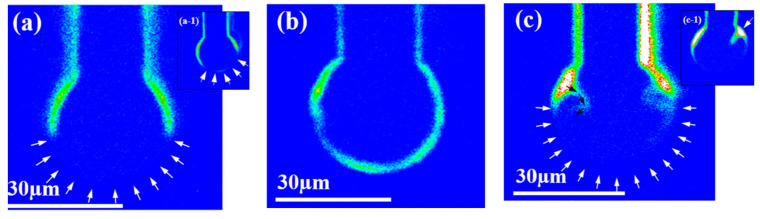
Pd distribution seen at the FAB cross-section with EPMA. (**a**–**c**) show the results for the FABs at low, medium, and high EFO current settings, respectively [38]. Copyright, 2018, Elsevier.

**Figure 28:**
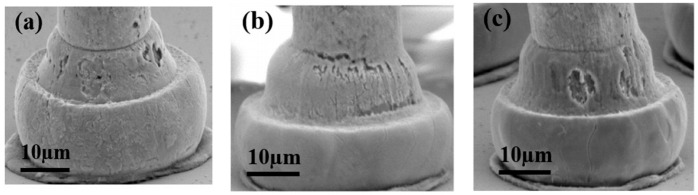
External corrosion of bonded balls after 500 h of HTST at 200 °C, as observed using SEM. (**a**–**c**) correspond to the low, medium, and high EFO current settings, respectively [38]. Copyright, 2018, Elsevier.

**Figure 29:**
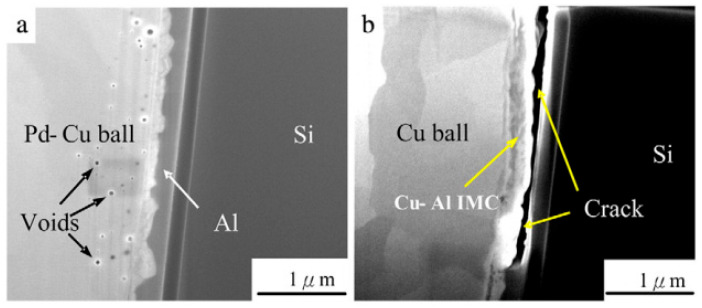
FIB study shows the cross-sectional microstructure of the interface between the ball bond and the Al pad after 336 h of PCT test. (**a**) Pd-coated Cu ball bond and (**b**) 4 N Cu ball bond [69]. Copyright, 2013, Elsevier.

Compared to bare Cu wire, PdCu wire has a high reliability. Uno et al. [10,13,97] used bare Cu wire and EX1 wire (PdCu wire, Pd layer thickness < 0.2 μm) to test and compare their bonding reliability. The results show that the life of EX1 wire in air is more than 90 days, and the life of bare Cu wire is 7 days. In the Pressure Cook Test (PCT) and unbias Highly Accelerated Stress Test (uHAST), EX1 wire has a better performance in bonding strength, tensile strain, and life extension, and the life of EX1 in uHAST is longer than that in PCT, and the shear strength after uHAST is more stable. Cheng et al. [16,38] observed the distribution of Pd on FAB by setting high, medium, and low EFO discharge currents, and then studied the effect of Pd distribution on bonding reliability. The results show that under low EFO discharge current, Pd was unevenly distributed in FAB (Figure 27). In addition, the “apple bite mark” indicating partial Pd coating on FAB also appeared (Figure 27a-1). Under the medium EFO discharge current, good Pd coverage is achieved on the FAB. (Figure 27b); under the high EFO discharge current setting, the “ vortex tunnel ” appears in the FAB (Figure 27c), and Pd is mainly concentrated in the neck area of the FAB (Figure 27c-1). Then, through the bonding test, the bonded sample was stored at a high temperature for 500 h, and its corrosion was observed (Figure 28). Under the high EFO discharge current and low EFO discharge current settings, the distribution of Pd on the bonding ball was incomplete, so the bonding ball was corroded seriously (Figure 28a,c). Under the medium EFO discharge current setting, the distribution of Pd was relatively uniform, and the corrosion was relatively slight (Figure 28b). It can be concluded that the uniform distribution of Pd on FAB can slow down the corrosion of the bonding ball, thereby improving the bonding reliability. Lin et al. [69] used bare Cu wires with a diameter of 20 μm and PdCu wires (with a Pd layer thickness of 100 nm), and conducted a comparative study of aging under PCT conditions for 336 h. They found that only a small amount of voids were formed at the bonding interface of PdCu wires (Figure 29a), and no cracks were observed; at the bonding interface of the Cu wires, obvious cracks were observed (Figure 29b).

**Figure 30:**
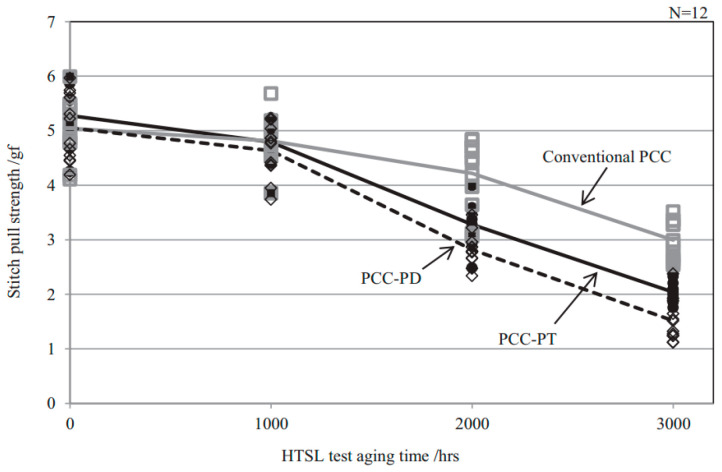
Change of stitch pull strength under HTSL test at 175 °C [98]. Copyright, 2021, Elsevier.

**Figure 31:**
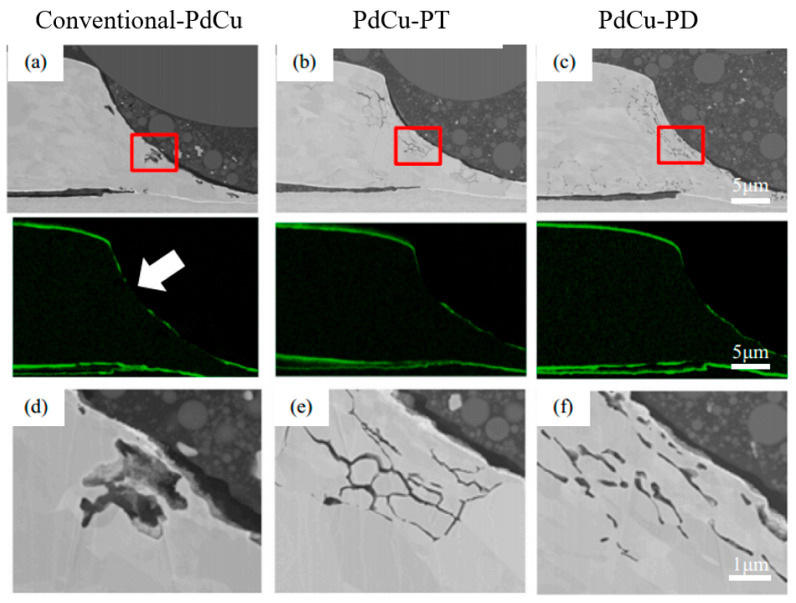
Cross-sectional SEM images (**a**–**c**) and corresponding Pd EDS mappings at the stitch bond area, and enlarged SEM images (**d**–**f**) for area indicated by area in (**a**–**c**) under HTSL test at 175 °C for 3000 h. [98]. Copyright, 2021, Elsevier.

**Figure 32:**
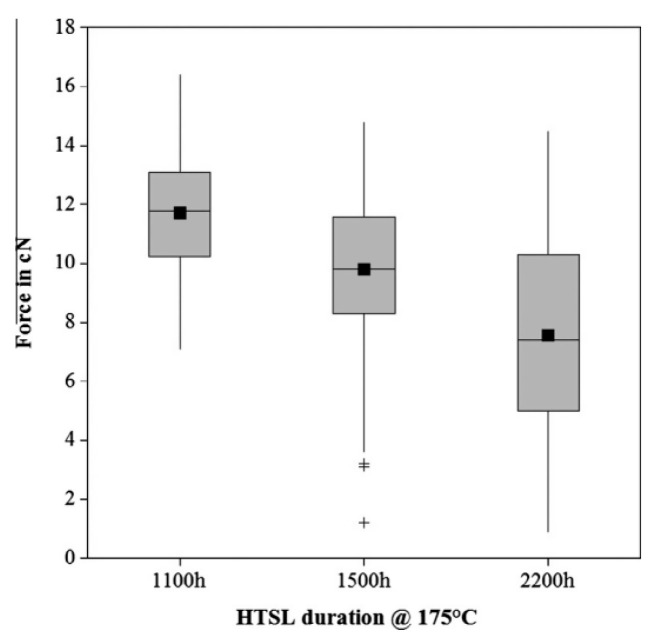
Box plot of bond pull values with the mode ‘‘broken stitch’’ [100]. Copyright, 2014, Elsevier.

**Figure 33:**
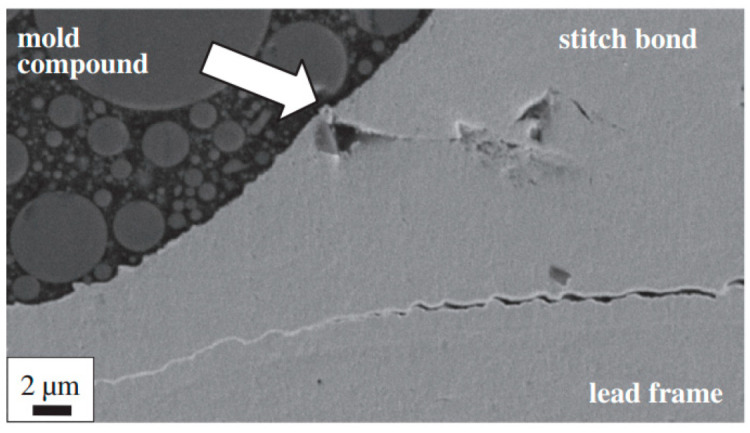
Cross sectional SEM micrograph of the molded PdCu wire stitch bonded on a pre-plated lead frame after 2200 h@175 °C HTSL. The arrow marks the location of the hillock on top of a void in the Cu core [100]. Copyright, 2014, Elsevier.

Some scholars have studied the stitch bond of PdCu wire and found that the rupture of the Pd layer is the main reason for the decrease of stitch bond strength. Qin et al. [44] studied the second bond using 18 μm PdCu wire and bare Cu wire, and they believe that the second bond consists of a sting bond and a tail bond. The tail bond strength is very important for the stable second bond process and is an indicator of the second bond assist rate (short tail rate). If the tail bond strength is too low, there will be a short tail. The experimental results show that the main difference between the PdCu wire and the bare Cu wire is the tail pull force. The tail pull force of the PdCu wire is much higher than that of the Cu wire, even more than 50%. Therefore, the PdCu wire has a lower short tail rate, which is one of the key reasons why the PdCu wire can be applied in the production of fine pitch. Eto et al. [98,99] studied the effect of adding new elements to the Cu core on the long-term reliability of PdCu wire under high-temperature conditions, and used traditional PdCu wire, PdCu-0.45 at. % Pd (PdCu-PD) wire, PdCu-0.27 at. % Pt (PdCu-PT) wire to test. The experimental results show that the bonding strength of the three bonding wires decreases gradually, and the stitch pull strength of PdCu-PD and PdCu-PT wires decreases faster than that of traditional PdCu wire (Figure 30). According to their analysis, the reason for the decrease of the stitch pull strength of the traditional PdCu wire is that the Pd layer is broken during bonding, so that the Cu matrix is exposed to the molding compound (Figure 31a,d), and the molding compound will decompose at high temperatures. Impurity (S, etc.) corrodes Cu; the decrease of stitch pull strength of PdCu-PD and PdCu-PT wires is due to intergranular corrosion, and there are network cracks on the stitch bond surface (Figure 31b,c,e,f). The added Pd and Pt are enriched near the grain boundary and the crack part, thereby preventing the corrosion from progressing to the grain, but at the same time accelerating the corrosion along the grain boundary. Krinke et al. [100] conducted a high-temperature storage life (HTSL) test using a 30 μm PdCu wire and found that the stitch pull strength decreased faster as the storage time increased (Figure 32). The reason is that the Pd layer is broken at the time of the stitch bond, which opens the diffusion path of Cu to the surface of Pd, and then forms a gap, and a hillock is formed above the gap (CuO, shown by the white arrow in Figure 33).

In summary, the appropriate amount of IMCs at the bonding interface is the key to improve the bonding strength. The Pd at the bonding interface can slow down the growth of Cu-Al IMCs, thereby improving the reliability of PdCu wires. The Pd on the surface of the bonding ball can prevent the bonding ball from being corroded by some halogen ions and improve the reliability of the PdCu wire in a humid environment. The stitch bond strength and bonding process window of the PdCu wire are better than those of bare Cu wire. The rupture of the Pd layer during stitch bond is the main reason for the decrease of bonding strength. Therefore, in the application process, we should optimize the test steps or improve the tool structure to avoid the rupture of the Pd layer.

## 5. Reliability of PdCu Wires

The reliability of metal bonding wires largely determines whether they can be widely used, and also determines the service life of electronic products. Therefore, it is necessary to evaluate the reliability of metal bonding wires by simulating the changes of temperature, humidity, and other factors in the actual environment through experiments. Pd belongs to the platinum group in the periodic table of elements. It is a noble metal with high chemical stability and has good adhesion to Cu. Therefore, the reliability of PdCu wire is considered to be derived from the anti-corrosion characteristics of Pd, and many scholars have also studied it.

**Figure 34:**
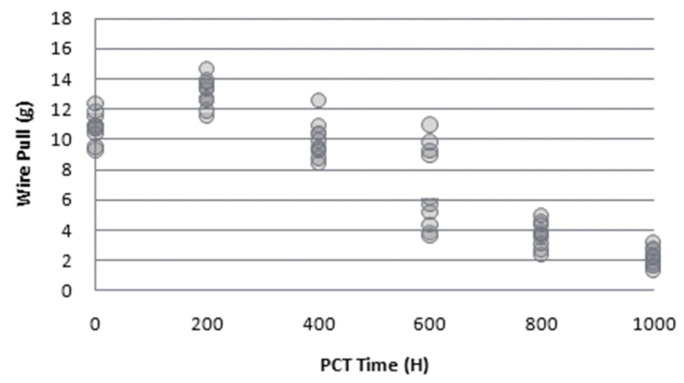
Pull force testing of bare Cu wire [102]. Reproduced with permission from ref. [102]; published by Electronics & Packaging, 2012.

**Figure 35:**
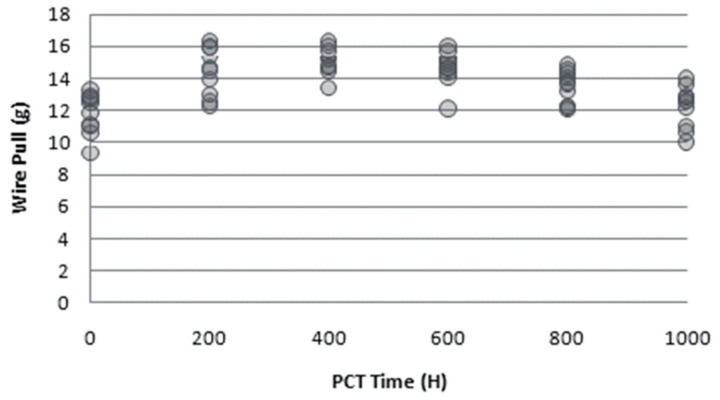
Pull force testing of PdCu wire [102]. Reproduced with permission from ref. [102]; published by Electronics & Packaging, 2012.

From the development history of metal bonding wires, Cu wire replaces Au wire, and PdCu wire replaces Cu wire, so some scholars have compared the reliability of these three wires. Chen [101] used 0.8 mil PdCu wire and bare Cu wire, selected silver-plated substrate as a bonding material, and carried out a bonding-strength test and a high-temperature storage reliability test (HTS) on the second bonding point of the bonding sample. In the bonding strength test, the tensile strength of the bare Cu wire is between 4.5–5.6, and the PdCu wire is between 7.2–7.9; in HTS, the bonding force of PdCu wire is gradually increasing, while that of bare Cu wire is decreasing in the initial stage, and then maintains a constant strength. Therefore, it can be fully demonstrated that the bonding strength of PdCu wire at the second bonding point is better than that of bare Cu wire, which reflects the high reliability of PdCu wire. Zhao [102] compared the reliability of PdCu wire and bare Cu wire by PCT test and pull force test. In PCT, due to the infiltration of water molecules into the epoxy resin of the plastic-encapsulated chip and ionization into H^+^ and HO^−^, coupled with the presence of halogen elements Br^+^ or Cl^−^, the entire environment exhibits weak acidity. In this environment, due to electrochemical corrosion, the IMCs at the edge of the bare Cu wire bonding point are first corroded, and the halogen elements gradually diffuse to the central area, then form craters, reduce the bonding strength, and finally the bonding fails, while no crater is found in the IMCs of the PdCu wire. The pull force test at different times of PCT showed that the pull force of the bare Cu wire was getting smaller and smaller (Figure 34), while the pull force of the PdCu wire did not decrease significantly (Figure 35). Lee et al. [68] found in the extended high-temperature storage life test that the interface crack line diffusion and IMC growth of PdCu wire were slower than those of bare Cu wire, and the reliability and second bonding strength of PdCu wire were higher than those of bare Cu wire. Gan et al. [103] used 0.8 mil PdCu wire and Au wire to cycle 2000 times in the temperature cycling test (TC, −40 °C–150 °C), stored in uHAST (130 °C, 85% RH) for 288 h and 1056 h, respectively, and then evaluated its reliability by measuring the pull force and shear strength of the bonded samples. The experimental results show that the pull force and shear strength of the two bonding wires decrease compared with the zero moment. However, compared with Au wire, the decrease of PdCu wire is smaller, which can also reflect that PdCu wire has higher reliability.

Reliability testing on molded units is probably the closest test to real-world application, because the pH value and chlorine content in the molding compound are the most important factors affecting the reliability of the Cu wire [104]. Stephan et al. [105] used green compounds (without halogen elements such as Br^+^ or Cl^−^) and non-green compounds to encapsulate the bonded samples, and studied the reliability of 20 μm PdCu wire in HTS, HAST, and PCT reliability tests. In HTS and HAST, for green compounds, PdCu wire performed well and no bonding failure occurred. For non-green compounds, PdCu wire can withstand up to 1000 h and 380 h tests, respectively. In the PCT, for the ball pull test, the initial bonding force of the PdCu wire was 9.2 g, and then remained at approximately 8 g on average until the end of the 384 h test; for the stitch pull test there was no degradation observed from 0 h up to the test’s end at 384 h. Uno et al. [106] used conventional resin (containing Br^+^) and green resin (without Br^+^) to encapsulate the bonded samples, and then studied the reliability of 25 μm PdCu wire and bare Cu wire in PCT and uHAST. The experimental data show that in PCT, for green resin, the shear strength of bare Cu wire begins to decrease from 40 mN to approximately 35 mN at 250 h, and reaches less than 20 mN at 400 h. The shear strength of PdCu wire remains stable above 600 h, and decreases slightly from 800 h to 1000 h. For traditional resins, the results are similar to those in green resins. In uHAST, for green resin, the shear strength of bare Cu wire decreased sharply within 100 h, while the shear strength of PdCu wire did not decrease within 1800 h. Therefore, regardless of the molding resin and the aging conditions of humidity and temperature, the humidity reliability of the PdCu wire is significantly higher than that of the bare Cu wire.

**Figure 36:**
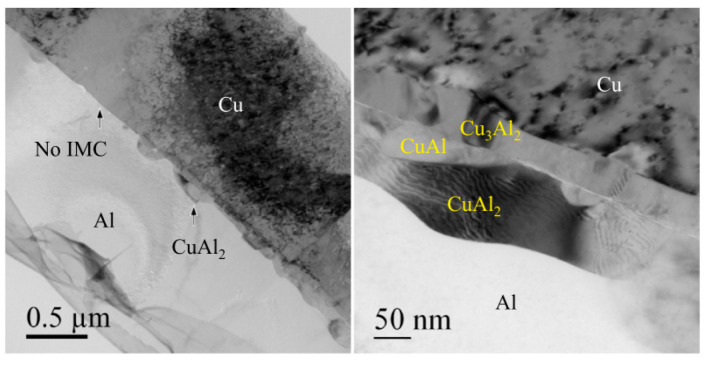
TEM images of the bonding interface under the bare Cu wire [17]. Copyright, 2019, Elsevier.

**Figure 37:**
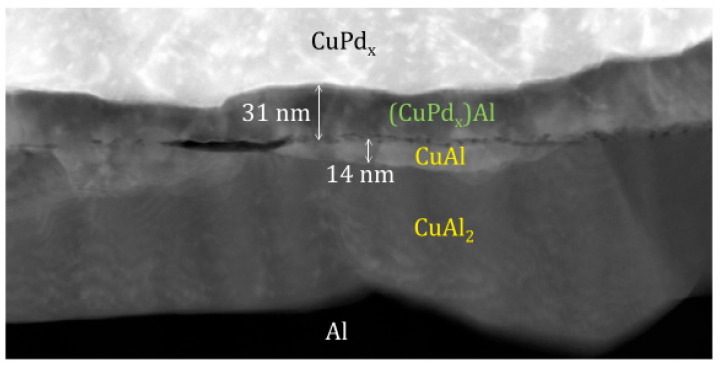
HAADF image of the bonding interface under the PdCu wire [17]. Copyright, 2019, Elsevier.

In IMCs, the corrosion of Cu-rich phase is often the main reason for the decrease of reliability. Qin et al. [16] found that the most Cu-rich IMCs under bare Cu wire is Cu_3_Al_2_ (Figure 36) and the most Cu-rich IMCs under PdCu wire is (CuPdx)Al (Figure 37) after uHAST using 20 μm bare Cu wire and PdCu wire. After analysis, it was found that the Pd layer promotes the formation of (CuPdx)Al and inhibits the formation of Cu-rich (CuPdx)_3_Al_2_, and the cathode/anode area ratio of (CuPdx)Al is lower than that of Cu_3_Al_2_, so the corrosion rate of (CuPdx)Al becomes slower, and the reliability of PdCu wire is higher than that of bare Cu wire.

**Figure 38:**
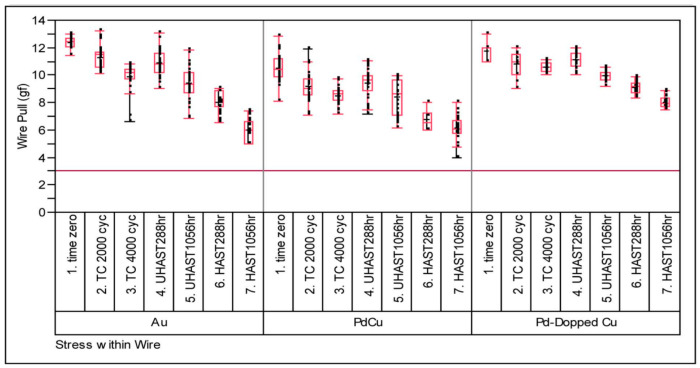
Post stresses wire pull strengths (g) of Au, PdCu, and Pd-doped Cu wires [107].

**Figure 39:**
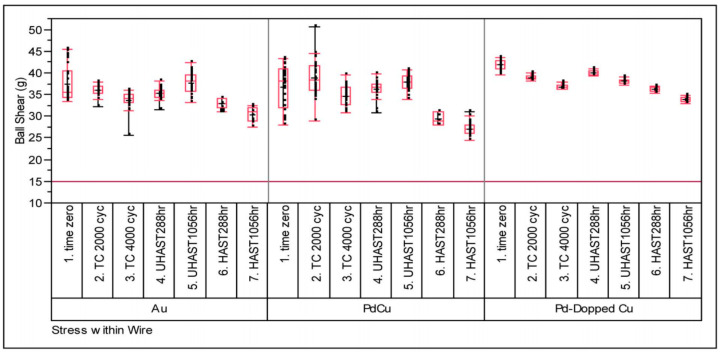
Post stresses ball shear strengths (g) of Au, PdCu, and Pd-doped Cu wires [107].

The Pd-doped Cu wire may have higher reliability than the Pd-coated Cu (PdCu) wire, because the Pd-doped Cu wire allows Pd to be better uniformly distributed in the FAB during the ball burning stage. Leong et al. [107] used 0.8 mil Pd-coated Cu (PdCu) wire, Pd-doped Cu wire and 4 N (purity 99.99%) Au wire to evaluate their reliability by measuring the pull force and shear strength of bonded samples in TC, UHAST, and HAST. The test results show that the three bonding wires far exceed the industry minimum pass standard (pull value: 3 g and ball shear value: 15 g). However, compared with Au wire and PdCu wire, the pull strength of Pd-doped Cu wire decreases slightly (Figure 38), and its shear strength value changes little and the degree of degradation is low (Figure 39).

In summary, PdCu wire has a high level of reliability, especially in high-temperature and high-humidity environments, which is the main reason why it can be widely used. However, PdCu wire is not a panacea for all problems. With the high integration and miniaturization of electronic devices, we may need better performance and higher reliability metal bonding wires (such as Pd-doped Cu wire), especially in some ultra-narrow spacing bonding.

## 6. Summaries

In this paper, the preparation and application of PdCu wire are reviewed. In the aspect of preparation, three kinds of Pd plating technologies, electroplating, electroless plating, and direct plating, are introduced. Electroplating and electroless plating will produce waste liquid and harmful substances in the production process, which will bring pressure to the ecological environment. The production process of direct plating is environmentally friendly and has no harmful substances, which can be called ‘green palladium plating‘. In terms of the surface quality of the coating, direct plating is superior to electroplating and electroless plating, and the cost of direct plating is only approximately 11% of electroplating and electroless plating. In terms of application, the factors affecting the distribution of Pd on FAB and the behavior of Pd at the bonding interface, the effect of Pd on the growth rate and reliability of IMCs, stitch bond and reliability of PdCu wire are analyzed. There are many factors affecting the distribution of Pd on FAB, such as EFO discharge current, EFO discharge time, EFO discharge electrode position, Pd layer thickness, environmental factors of aging test, type of PdCu wire, etc. Among them, EFO discharge current has a significant effect. The low EFO discharge current causes Pd to be concentrated in the neck of the FAB, and the high EFO discharge current causes some Pd to be injected into the FAB due to the turbulence of Cu. Under the medium EFO discharge current, Pd can be relatively evenly distributed on the surface of the FAB. Therefore, in the test, the appropriate EFO current should be selected according to the wire diameter. With the vertex of FAB as the center, approximately 44.5% of the spherical surface area will participate in the formation of the bonding interface, so Pd in this area will exist at the bonding interface, but the position of Pd at the bonding interface will move to the periphery of the bonding interface with the increase of aging time, and form a Pd-rich layer. Pd slows down the growth rate of Cu-Al IMCs at the bonding interface thus making the thickness of IMCs thinner. The rupture of the Pd layer can cause the Cu substrate to be exposed under the molded plastic, causing it to corrode. Therefore, the rupture of the Pd layer is the main reason for the decrease in stitch bond strength. The reliability of PdCu wire is higher than that of bare Cu wire and Au wire, especially in high-temperature and high-humidity environments.

## 7. Future Prospects

With the progress of science and technology, our lives consist of more and more digital and information technology, and these are inseparable from the development of electronic products. Nowadays, many electronic products are developing towards high integration and miniaturization, which requires us to develop a bonding wire with ultra-fine, ultra-long, ultra-precision, high conductivity, high-thermal conductivity, and high elongation, and PdCu wire basically meets the above requirements. In addition, the new infrastructure construction mentioned in the 2020 State Council Work Report mainly includes 5 G, new energy vehicle charging piles, artificial intelligence, industrial Internet, and other fields. The development of these fields will bring opportunities and challenges regarding the application of PdCu wires. Therefore, we should study the performance of PdCu wires more deeply on the basis of previous studies and explore newer preparation processes and palladium-plating technologies as much as possible to improve the reliability of PdCu wires.

## Data Availability

Not applicable.

## References

[1] Zhong Z.W. (2011). Overview of wire bonding using copper wire or insulated wire. Microelectron. Reliab..

[2] Charles J., Gunasekaran M., Malliah R. (2011). Factors affecting the long-term stability of Cu/Al ball bonds subjected to standard and extended high temperature storage. Microelectron. Reliab..

[3] Qin I.W., Chylak B., Clauberg H., Shah A., Foley J. (2012). Ball Bond Process Optimization with Cu and Pd-Coated Cu Wire. ECS Trans..

[4] Chauhan P.S., Choubey A., Zhong Z.W., Pecht M.G. (2014). Copper Wire Bonding.

[5] Abdul R.D., Tan C.W. (2003). Mechanical and Electrical Properties of Au-Al and Cu-Al Intermetallics Layer at Wire Bonding Interface. J. Electron. Packag..

[6] Cho J.-H., Rollett A.D., Cho J.-S., Papk Y.-J., Moon J.-T., OH K.H. (2006). Investigation of recrystallization and grain growth of copper and gold bonding wires. Metall. Mater. Trans. A.

[7] Schneider-Ramelow M., Ehrhardt C. (2016). The reliability of wire bonding using Ag and Al. Microelectron. Reliab..

[8] Nguyen L.T., Mcdonald D. (1995). Optimization of copper wire bonding on Al-Cu metallization. IEEE Trans. Compon. Packag. Manuf. Technol..

[9] Murali S., Srikanth N., Vath III C.J. (2003). Grains, deformation substructures, and slip bands observed in thermosonic copper ball bonding. Mater. Charact..

[10] Uno T. (2011). Bond reliability under humid environment for coated copper wire and bare copper wire. Microelectron. Reliab..

[11] Tan C.W., Daud A.R., Yarmo M.A. (2002). Corrosion study at Cu–Al interface in microelectronics packaging. Appl. Surf. Sci..

[12] Kaimori S., Nonaka T., Mizoguchi A. (2006). Development of ‘Hybrid Bonding Wire’. SEI Tech. Rev..

[13] Uno T. (2011). Enhancing bondability with coated copper bonding wire. Microelectron. Reliab..

[14] Zhou H., Zhang Y., Cao J., Su C., Li C., Chang A., An B. (2023). Research Progress on Bonding Wire for Microelectronic Packaging. Micromachines.

[15] Kaimori S., Nonaka T., Mizoguchi A. (2006). The Development of Cu Bonding Wire with Oxidation-Resistant Metal Coating. IEEE Trans. Adv. Packag..

[16] Cheng P.-Y., Lai P.-Y., Ye Z.-J., Hesieh C.-L., Ye J.-M. (2017). Effect of Pd distribution on Pd-plated Cu wire using different electronic flame off settings. J. Mater. Sci. Mater. Electron..

[17] Qin W., Anderson T., Chang G. (2019). Mechanism to improve the reliability of copper wire bonding with palladium-coating of the wire. Microelectron. Reliab..

[18] Koh W., Lee T.K., Ng H.S., Goh K.S. Investigation of palladium coverage on bonded balls of palladium-coated copper wires. Proceedings of the IEEE 2011 12th International Conference on Electronic Packaging Technology and High Density Packaging.

[19] Xu H., Qin I., Clauberg H., Chylak B., Acoff V.L. (2013). Behavior of palladium and its impact on intermetallic growth in palladium-coated Cu wire bonding. Acta Mater..

[20] Stephan D., Chew E., Yeung J., Milke E. Impact of palladium to the interfacial behavior of palladium coated copper wire on aluminium pad metallization during high temperature storage. Proceedings of the IEEE 2011 IEEE 13th Electronics Packaging Technology Conference.

[21] Uno T., Kimura K., Yamada T. Surface-enhanced copper bonding wire for LSI and its bond reliability under humid environment. Proceedings of the IEEE 2009 European Microelectronics and Packaging Conference.

[22] England L., Eng S.T., Liew C., Lim H.H. (2010). Cu wire bond parameter optimization on various bond pad metallization and barrier layer material schemes. Microelectron. Reliab..

[23] Gam S.-A., Kim H.-J., Cho J.-S., Park Y.-J., Moon J.-T., Paik K.-W. (2006). Effects of Cu and Pd Addition on Au Bonding Wire/Al Pad Interfacial Reactions and Bond Reliability. J. Electron. Mater..

[24] Gan C.L., Hashim U. (2013). Reliability Assessment and Activation Energy Study of Au and Pd-Coated Cu Wires Post High Temperature Aging in Nanoscale Semiconductor Packaging. J. Electron. Packag..

[25] Kong Y.N. (2013). The Research on Manufacturing Process and Properties of Palladium Coated Copper Wire. Master’s Thesis.

[26] Cao R.-P., Xiao S.-M. (2004). Study on Technology of Palladium Plating. Surf. Technol..

[27] Kang F.F., Yang G.X., Kong J.W., Dao P., Wu Y.J., Zhang K.H. (2011). The Development Trend of Palladium–plated Bonding Copper Wire. Mater. Rev..

[28] Cao J., Fan J.L., Gao W.B. (2015). Investigation of copper direct coating Pd technology and bonding properties. Mater. Sci. Technol..

[29] Zhang T.-T., Zhao L.-L., Wan C.-Y., He Y.-Q., Xu B. (2015). Study on process of electroless palladium plating on copper. Electroplat. Finish..

[30] Zhou X.G., Du L.M., Xiang C.H., Su H.F., Chen B. (2016). A Chemically Plated Palladium Copper Bonding Wire and Its Preparation Method. China Patent.

[31] Zhang T.-T. (2016). Improvement of Stability of Copper Wire Bonding. Master’s Thesis.

[32] Ding Y.-T., Cao J., Hu Y., Kou S.-Z., Xu G.-J. (2007). Study on Effect Factor for Preparation Process of Single Crystal Copper Bonding Wire. Foundry Technol..

[33] Ding Y.T., Kong Y.N., Cao J., Hu Y., Sun G. (2013). Research on Producing Process and Performance of Pd-coated Copper Wire. Foundry Technol..

[34] Song K.-X., Zhou Y.-J., Mi X.-J., Xiao Z., Cao J., Ding Y.-T., Wu B.-A., Feng C.-L., Li Z., Chen D.-B. (2020). Preparation, microstructure and properties of copper based wire. Trans. Nonferrous Met. Soc. China.

[35] Yauw O., Clauberg H., Lee K.F., Shen L., Chylak B. Wire bonding optimization with fine copper wire for volume production. Proceedings of the IEEE 2010 12th Electronics Packaging Technology Conference.

[36] Tang L.J., Ho H.M., Koh W., Zhang Y.J., Goh K.S., Huang C.S., Yu Y.T. Pitfalls and solutions of replacing gold wire with palladium coated copper wire in IC wire bonding. Proceedings of the IEEE 2011 IEEE 61st Electronic Components and Technology Conference (ECTC).

[37] Clauberg H., Chylak B., Wong N., Yeung J., Milke E. Wire bonding with Pd-coated copper wire. Proceedings of the IEEE 2010 IEEE CPMT Symposium Japan.

[38] Cheng P.Y., Lai P.Y., Ye J.M., Chen T.C., Hsieh C.L. (2018). High temperature storage reliability of palladium coated copper wire in different EFO current settings. Microelectron. Reliab..

[39] Lim A.B.Y., Chang A.C.K., Lee C.X., Yauw O., Chylak B., Chen Z. (2013). Palladium-Coated and Bare Copper Wire Study for Ultra-Fine Pitch Wire Bonding. ECS Trans..

[40] Lim A.B.Y., Chang A.C.K., Yauw O., Chylak B., Gan C.L., Chen Z. (2014). Ultra-fine pitch palladium-coated copper wire bonding: Effect of bonding parameters. Microelectron. Reliab..

[41] Du Y.H., Liu Z.Q., Ji H.J., Li M.Y., Wen M. (2018). The mechanism of Pd distribution in the process of FAB formation during Pd-coated Cu wire bonding. J. Mater. Sci. Mater. Electron..

[42] Ly N., Xu D.E., Song W.H., Mayer M. (2015). More uniform Pd distribution in free-air balls of Pd-coated Cu bonding wire using movable flame-off electrode. Microelectron. Reliab..

[43] Qin I., Xu H., Clauberg H., Cathcart R., Acoff V., Chylak B., Huynh C. Wire bonding of Cu and Pd coated Cu wire: Bondability, reliability, and IMC formation. Proceedings of the IEEE 2011 IEEE 61st Electronic Components and Technology Conference (ECTC).

[44] Subramanian P.R., Simmons J.P. (1990). Phase Equilibria in the Vicinity of the DO22Al3Nb Composition in the AI-Nb-W, AI-Nb-Co, AI-Nb-Pt, AND AI-Nb-Ag Systems. Scr. Metall. Mater..

[45] Yeung J., Xu H., Chew E. Effect of palladium on copper aluminide intermetallic growth in palladium copper bonding wire. Proceedings of the IEEE 2012 13th International Conference on Electronic Packaging Technology & High Density Packaging.

[46] Cao J., Fan J.L., Xue T.L. (2014). Investigation of copper coating Pd wire properties and bonding quality. Mater. Sci. Technol..

[47] Tang L.J., Ho H.M., Zhang Y.J., Lee Y.M., Lee C.W. Investigation of Palladium Distribution on the Free Air Ball of Pd-coated Cu wire. Proceedings of the IEEE 2010 12th Electronics Packaging Technology Conference.

[48] Du Y.H. (2020). Influence of Palladium Addition on the Reliability of Copper Wire Bond. Ph.D. Thesis.

[49] Hang C.J., Wang C.Q., Mayer M., Tian Y.H., Zhou Y. (2008). Growth behavior of Cu/Al intermetallic compounds and cracks in copper ball bonds during isothermal aging. Microelectron. Reliab..

[50] Yoshitaka T., Jian L., Russell S.W., Mayer J.W. (1992). Thermal and ion beam induced thin film reactions in Cu-Al bilayers. Nucl. Instrum. Methods Phys. Res. Sect. B.

[51] Ratchev P., Stoukatch S., Swinnen B. (2005). Mechanical reliability of Au and Cu wire bonds to Al, Ni/Au and Ni/Pd/Au capped Cu bond pads. Microelectron. Reliab..

[52] Onuki J., Koizumi M., Araki I. (1987). Investigation of the Reliability of Copper Ball Bonds to Aluminum Electrodes. IEEE Trans. Compon. Hybrids Manuf. Technol..

[53] Murali S., Srikanth N., Vath C.J. (2003). An analysis of intermetallics formation of gold and copper ball bonding on thermal aging. Mater. Res. Bull..

[54] Kim S.H., Park J.W., Hong S.J., Moon J.T. The interface behavior of the Cu-Al bond system in high humidity conditions. Proceedings of the IEEE 2010 12th Electronics Packaging Technology Conference.

[55] Kim H.J., Lee J.Y., Paik K.W., Koh K.W., Won J., Choe S., Lee J., Moon J.T., Park Y.J. (2003). Effects of Cu/Al Intermetallic Compound (IMC) on Copper Wire and Aluminum Pad Bondability. IEEE Trans. Compon. Packag. Manuf. Technol..

[56] Chen C.Y., Hwang W.S. (2007). Effect of Annealing on the Interfacial Structure of Aluminum-Copper Joints. Mater. Trans..

[57] Lim A.B.Y., Long X., Shen L., Chen X., Ramanujan R.V., Gan C.L., Chen Z. (2015). Effect of palladium on the mechanical properties of Cu–Al intermetallic compounds. J. Alloys Compd..

[58] Tavassoli S., Abbasi M., Tahavvori R. (2016). Controlling of IMCs layers formation sequence, bond strength and electrical resistance in Al-Cu bimetal compound casting process. Mater. Des..

[59] Xu H., Liu C., Silberschmidt V.V., Chen Z., Wei J., Sivakumar M. (2011). Effect of bonding duration and substrate temperature in copper ball bonding on aluminium pads: A TEM study of interfacial evolution. Microelectron. Reliab..

[60] Lim A.B.Y., Neo W.J., Yauw O., Chylak B., Gan C.L., Chen Z. (2016). Evaluation of the corrosion performance of Cu–Al intermetallic compounds and the effect of Pd addition. Microelectron. Reliab..

[61] Park H.W., Lee S.J., Cho D.C., Lee S.H., Kim J.K., Lee J.H., Jung S.K., Narm H.S., Hsu P., Low S. (2018). Behavior of Au and Pd and the effects of these metals on IMCs in Pd-Au-coated copper wire. Microelectron. Reliab..

[62] Na S.H., Hwang T.K., Park J.S., Kim J.Y., Yoo H.Y., Lee C.H. Characterization of intermetallic compound (IMC) growth in Cu wire ball bonding on Al pad metallization. Proceedings of the IEEE 2011 IEEE 61st Electronic Components and Technology Conference (ECTC).

[63] Yamaji Y., Hori M., Ikenosako H., Oshima Y., Suda T., Umeki A., Kandori M., Oida M., Goto H., Katsumata A. IMC study on Cu wirebond failures under high humidity conditions. Proceedings of the IEEE 2011 IEEE 13th Electronics Packaging Technology Conference.

[64] Lim A.B.Y., Boothroyd C.B., Yauw O., Chylak B., Gan C.L., Chen Z. (2016). Interfacial evolution and bond reliability in thermosonic Pd coated Cu wire bonding on aluminum metallization: Effect of palladium distribution. Microelectron. Reliab..

[65] Abe H., Kang D.C., Yamamoto T., Yagihashi T., Endo Y., Saito H., Horie T., Tamate H., Ejiri Y., Watanabe N. Cu wire and Pd-Cu wire package reliability and molding compounds. Proceedings of the IEEE 2012 IEEE 62nd Electronic Components and Technology Conference.

[66] Lee C.C.S., Tran T., Boyne D., Higgins L., Mawer A. Copper versus palladium coated copper wire process and reliability differences. Proceedings of the IEEE 2014 IEEE 64th Electronic Components and Technology Conference (ECTC).

[67] Schneider-Ramelow M., Geißler U., Schmitz S., Grübl W., Schuch B. (2013). Development and Status of Cu Ball/Wedge Bonding in 2012. J. Electron. Mater..

[68] Lu Y.H., Wang Y.W., Appelt B.K., Lai Y.S., Kao C.R. Growth of CuAl intermetallic compounds in Cu and Cu(Pd) wire bonding. Proceedings of the IEEE 2011 IEEE 61st Electronic Components and Technology Conference (ECTC).

[69] Lin Y.W., Ke W.B., Wang R.Y., Wang I.S., Chiu Y.T., Lu K.C., Lin K.L., Lai Y.S. (2013). The influence of Pd on the interfacial reactions between the Pd-plated Cu ball bond and Al pad. Surf. Coat. Technol..

[70] Xu H., Qin I., Shan A., Clauberg H., Chylak B., Acoff V.L. TEM study on interface of palladium coated copper wire bonding on aluminum metallization. Proceedings of the IEEE 2012 13th International Conference on Electronic Packaging Technology & High Density Packaging.

[71] Philofsky E. (1970). Intermetallic Formation in Gold-Aluminum Systems. Solid-State Electron..

[72] Jang G.Y., Duh J.G., Takahashi H., Su D. (2006). Solid-State Reaction in an Au Wire Connection with an Al-Cu Pad During Aging. J. Electron. Mater..

[73] Kim H.G., Lee T.W., Jeong E.K., Kim W.Y., Lim S.H. (2011). Effects of alloying elements on microstructure and thermal aging properties of Au bonding wire. Microelectron. Reliab..

[74] Gan C.L., Ng E.K., Chan B.L., Classe F.C., Kwuanjai T., Hashim U. (2013). Wearout Reliability and Intermetallic Compound Diffusion Kinetics of Au and PdCu Wires Used in Nanoscale Device Packaging. J. Nanomater..

[75] Breach C.D., Wulff F. (2004). New observations on intermetallic compound formation in gold ball bonds: General growth patterns and identification of two forms of Au 4 Al. Microelectron. Reliab..

[76] Lin Y.W., Wang R.Y., Ke W.B., Wang I.S., Chiu Y.T., Lu K.C., Lin K.L., Lai Y.S. (2012). The Pd distribution and Cu flow pattern of the Pd-plated Cu wire bond and their effect on the nanoindentation. Mater. Sci. Eng. A.

[77] Chen J., Lai Y.S., Wang Y.W., Kao C.R. (2011). Investigation of growth behavior of Al–Cu intermetallic compounds in Cu wire bonding. Microelectron. Reliab..

[78] Drozdov M., Gur G., Atzmon Z., Kaplan W.D. (2008). Detailed investigation of ultrasonic Al–Cu wire-bonds: II. Microstructural evolution during annealing. J. Mater. Sci..

[79] Funamizu Y., Watanabe K. (1971). Interdiffusion in the Al–Cu System. Trans. Jpn. Inst. Met..

[80] Kah P., Vimalraj C., Martikainen J., Suoranta R. (2015). Factors influencing Al-Cu weld properties by intermetallic compound formation. Int. J. Mech. Mater. Eng..

[81] Du Y.H., Wen M., Ji H.J., Li M.Y., Liu Z.Q. (2019). Effects of Pd addition on the interfacial reactions between Cu and Al during ultrasonic welding. J. Mater. Sci. Mater. Electron..

[82] Liu X.J., Ohnuma I., Kainuma R., Ishida K. (1998). Phase equilibria in the Cu-rich portion of the Cu–Al binary system. J. Alloys Compd..

[83] Tan Y.Y., Yang Q.L., Sim K.S., Sun L.T., Wu X. (2015). Cu–Al intermetallic compound investigation using ex-situ post annealing and in-situ annealing. Microelectron. Reliab..

[84] Xu H., Liu C., Silberschmidt V.V., Pramana S.S., White T.J., Chen Z., Acoff V.L. (2011). Behavior of aluminum oxide, intermetallics and voids in Cu–Al wire bonds. Acta Mater..

[85] Kim H.G., Kim S.M., Lee Y.J. (2014). Microstructural evaluation of interfacial intermetallic compounds in Cu wire bonding with Al and Au pads. Acta Mater..

[86] Gueydan A., Domengès B., Hug E. (2014). Study of the intermetallic growth in copper-clad aluminum wires after thermal aging. Intermetallics.

[87] Drozdov M., Gur G., Atzmon Z., Kaplan W.D. (2008). Detailed investigation of ultrasonic Al–Cu wire-bonds: I. Intermetallic formation in the as-bonded state. J. Mater. Sci..

[88] Xu H., Liu C., Silberschmidt V.V., Chen Z. TEM Microstructural Analysis of As-bonded Copper Ball Bonds on Aluminum Metallization. Proceedings of the IEEE 2008 10th Electronics Packaging Technology Conference.

[89] Xu H., Liu C., Silberschmidt V.V., Pramana S.S., White T.J., Chen Z. (2009). A re-examination of the mechanism of thermosonic copper ball bonding on aluminium metallization pads. Scr. Mater..

[90] Guo Y.J., Liu G.W., Jin H.Y., Shi Z.Q., Qiao G.J. (2011). Intermetallic phase formation in diffusion-bonded Cu/Al laminates. J. Mater. Sci..

[91] Eto M., Araki N., Yamada T., Sugiyama M., Fujimoto S. (2021). Influence of post-bonding heating process on the long-term reliability of Cu/Al contact. Microelectron. Reliab..

[92] Gan C.L., Hashim U. (2015). Evolutions of bonding wires used in semiconductor electronics: Perspective over 25 years. J. Mater. Sci. Mater. Electron..

[93] Lee C.C., Higgins L.M. Challenges of Cu wire bonding on low-k/Cu wafers with BOA structures. Proceedings of the IEEE 2010 Proceedings 60th Electronic Components and Technology Conference (ECTC).

[94] Hiew P.F., Au Y.K., Eu P.L. Development and qualification of copper wire bond process for automotive applications. Proceedings of the IEEE 2012 14th International Conference on Electronic Materials and Packaging (EMAP).

[95] Tran T.A., Lee C.C., Mathew V., Higgins L. Copper wire bonding on low-k/copper wafers with Bond Over Active (BOA) structures for automotive customers. Proceedings of the IEEE 2011 IEEE 61st Electronic Components and Technology Conference (ECTC).

[96] Boettcher T., Rother M., Liedtke S., Ullrich M., Bollmann M., Pinkernelle A., Gruber D., Funke H.J., Kaiser M., Lee K. On the intermetallic corrosion of Cu-Al wire bonds. Proceedings of the IEEE 2010 12th Electronics Packaging Technology Conference.

[97] Uno T., Terashima S., Yamada T. Surface-enhanced copper bonding wire for LSI. Proceedings of the IEEE 2009 59th Electronic Components and Technology Conference.

[98] Eto M., Araki N., Yamada T., Sugiyama M., Fujimoto S. (2021). Microstructural characterization of alloyed palladium coated copper wire under high temperature. Microelectron. Reliab..

[99] Eto M., Araki N., Yamada T., Klengel S., Petzold M., Sugiyama M., Fujimoto S. (2020). Effects of alloying elements in high reliability copper wire bond material for high temperature applications. Microelectron. Reliab..

[100] Krinke J.C., Dragicevic D., Leinert S., Friess E., Glueck J. (2014). High temperature degradation of palladium coated copper bond wires. Microelectron. Reliab..

[101] Chen X. (2014). A comparative study of palladium-plated copper wire and bare copper wire bonding in IC packaging. Silicon Val..

[102] Zhao J. (2012). Discussion the Characteristics of Coating Pd Copper Wire Bonding. Electron. Packag..

[103] Gan C.L., Ng E.K., Chan B.L., Kwuanjai T., Jakarin S., Hashim U. Wearout reliability study of Cu and Au wires used in flash memory fine line BGA package. Proceedings of the IEEE 2012 7th International Microsystems, Packaging, Assembly and Circuits Technology Conference (IMPACT).

[104] Su P., Seki H., Ping C., Zenbutsu S.I., Itoh S., Huang L., Liao N., Liu B., Chen C., Tai W. An evaluation of effects of molding compound properties on reliability of Cu wire components. Proceedings of the IEEE 2011 IEEE 61st Electronic Components and Technology Conference (ECTC).

[105] Stephan D., Wulff F.W., Milke E. Reliability of palladium coated copper wire. Proceedings of the IEEE 2010 12th Electronics Packaging Technology Conference.

[106] Uno T., Yamada T. Improving humidity bond reliability of copper bonding wires. Proceedings of the IEEE 2010 Proceedings 60th Electronic Components and Technology Conference (ECTC).

[107] Leong G.C., Uda H. (2013). Comparative Reliability Studies and Analysis of Au, Pd-Coated Cu and Pd-Doped Cu Wire in Microelectronics Packaging. PLoS ONE.

